# Low-Zone IL-2 Signaling: Fusion Proteins Containing Linked CD25 and IL-2 Domains Sustain Tolerogenic Vaccination *in vivo* and Promote Dominance of FOXP3^+^ Tregs *in vitro*

**DOI:** 10.3389/fimmu.2020.541619

**Published:** 2020-09-23

**Authors:** Kayla B. DeOca, Cody D. Moorman, Brandon L. Garcia, Mark D. Mannie

**Affiliations:** Department of Microbiology and Immunology, Brody School of Medicine, East Carolina University, Greenville, NC, United States

**Keywords:** FOXP3^+^ Tregs, IL-2, CD25, EAE/MS, tolerance, neuroimmunology

## Abstract

Low-zone IL-2 signaling is key to understanding how CD4^+^ CD25^high^ FOXP3^+^ regulatory T cells (Tregs) exhibit dominance and overgrow conventional effector T cells (Tcons) that typically express lower levels of the IL-2 receptor alpha chain (i.e., CD25). Thus, modalities such as low-dose IL-2 or IL-2/anti-IL-2 antibody complexes have been advanced in the clinic to selectively expand Treg populations as a treatment for chronic inflammatory autoimmune diseases. However, more effective reagents that efficiently lock IL-2 signaling into a low signaling mode are needed to validate and exploit the low-zone IL-2 signaling niche of Tregs. This study focuses on CD25-IL2 and IL2-CD25 fusion proteins (FPs) that were approximately 32 and 320-fold less potent than IL-2. These FPs exhibited transient binding to transmembrane CD25 on human embryonic kidney (HEK) cells, had partially occluded IL-2 binding sites, and formed higher order multimeric conformers that limited the availability of bioactive IL-2. These FPs exhibited broad bell-shaped concentration ranges that favored dominant Treg outgrowth during continuous culture and were used to derive essentially pure long-term Treg monocultures (∼98% Treg purity). FP-induced Tregs had canonical Treg suppressive activity in that these Tregs suppressed antigen-specific proliferative responses of naïve CD4^+^ T cells. The *in vivo* administration of CD25-IL2/Alum elicited robust increases in circulating Tregs and selectively augmented CD25 expression on Tregs but not on Tcons. A single injection of a Myelin Oligodendrocyte Glycoprotein (MOG35-55)-specific tolerogenic vaccine elicited high levels of circulating MOG-specific Tregs *in vivo* that waned after 2–3 weeks, whereas boosting with CD25-IL2/Alum maintained MOG-specific CD25^high^ Tregs throughout the 30-day observation period. However, these FPs did not antagonize free monomeric IL-2 and lacked therapeutic efficacy in experimental autoimmune encephalomyelitis (EAE). In conclusion, these data reveal that CD25-IL2 FPs can be used to select essentially pure long-term lines of FOXP3^+^ CD25^high^ Tregs. This study also shows that CD25-IL2 FPs can be administered *in vivo* in synergy with tolerogenic vaccination to maintain high circulating levels of antigen-specific Tregs. Because tolerogenic vaccination and Treg-based adoptive immunotherapy are limited by gradual waning of Tregs, these FPs have potential utility in sustaining tolerogenic Treg responses *in vivo*.

## Introduction

FOXP3^+^ regulatory T cells (Tregs) are critical mediators of immune homeostasis that preempt chronic inflammatory syndromes across a wide gamut of autoimmune and hypersensitivity diseases (1–4). These FOXP3^+^ CD25^high^ Tregs are essential for maintenance of self-tolerance because loss-of-function mutations in *Foxp3* cause an early-onset, lethal autoimmune disorder known as immunodysregulation polyendocrinopathy enteropathy X-linked syndrome (IPEX) in humans and scurfy in mice (5, 6). Cardinal hallmarks of the FOXP3^+^ Treg lineage include higher expression levels of surface CD25 and exquisite sensitivity to low doses of IL-2 coupled with a lack of intrinsic IL-2 productive capacity (7, 8). Notably, Treg and Tcon subsets differ both in CD25 expression levels and in expression of the PI(3)K inhibitor PTEN, which is preferentially expressed in Tregs rather than Tcons (9–11). In IL-2-limited environments, Tregs dominate because constitutive expression of a CD25^high^ phenotype allows Tregs to monopolize the limited pool of paracrine IL-2 while depriving Tcons of the IL-2 needed to sustain effector function and survival, thereby extinguishing the autoimmune response (12). In IL-2-replete environments, Tcons dominate IL-2 driven expansion because the lack of PTEN in Tcons enables full IL-2 signaling via the PI(3)K pathway whereas Tregs have more limited IL-2 dependent growth due to PTEN-mediated repression of the PI(3)K pathway. These findings support the concept that low-zone vs high-zone IL-2 concentrations favor dominant outgrowth of suppressive Tregs vs effector Tcons, respectively.

The goal of Treg-based therapeutics is to reconstitute ‘holes in the Treg repertoire’ and thereby reverse chronic inflammatory autoimmune disease (13, 14). The derivation of novel Treg-based therapeutics is contingent upon our understanding of the Treg niche, which is defined by the TCR antigen recognition properties and the cytokine microenvironments that enable Treg dominance with the consequent establishment of an anti-inflammatory homeostatic environment. To exploit the Treg antigenic niche *in vivo*, tolerogenic vaccines may be optimized by incorporating self-antigens that are recognized with intermediate TCR recognition efficiencies (14–21). Conversely, vaccines incorporating antigens that are recognized with high TCR recognition efficiencies are optimal for induction of immunogenic Tcon responses (21–23). To exploit the Treg cytokine niche *in vivo*, low-dose IL-2 therapies or administration of IL-2/anti-IL-2 antibody complexes are used to selectively create low IL-2 concentrations in local environments that are optimal for Treg dominance (24–26). To exploit the Treg cytokine niche *in vitro*, the immunosuppressive drug Rapamycin is used to limit otherwise dominant Tcon outgrowth in the expansion cultures that are needed to stage Treg-based adoptive immunotherapy (27–29).

The Treg cytokine niche is also the basis for developing alternative technologies to advance Treg-based adoptive immunotherapy protocols. Addition of neutralizing anti-CD25 single-chain variable fragments or monoclonal antibodies (mAbs) to murine IL-2-stimulated Treg/Tcon cultures enabled dominant Treg outgrowth and establishment of continuous stable FOXP3^+^ Treg lines (30). In contrast, when propagated in the presence of IL-2 alone, mixed Treg/Tcon cultures exhibited dominant outgrowth of Tcons. The interpretation is that inhibitory anti-CD25 mAb preferentially blocked Tcons, which expressed a CD25^low–intermediate^ phenotype, but only partially blocked IL-2 stimulated expansion of Tregs, because the CD25^high^ Treg phenotype conferred resistance to this inhibitory mAb. This combination of high neutralizing anti-CD25 mAb concentrations coupled with low IL-2 concentrations enabled the derivation of continuous, long-term Treg lines comprised of CD25^high^ FOXP3^+^ MOG-specific Tregs. When activated and transferred into recipient mice, these TGF-β-induced Tregs inhibited disease in both prophylactic and therapeutic models of EAE (30). Overall, these findings validate low-zone IL-2 signaling as the key for Treg expansion technologies.

The principles underlying low-dose IL-2 therapy may provide leads for improved technologies for autologous Treg-based adoptive immunotherapy and tolerogenic vaccination. A central consideration is that Tregs specific for a relevant tissue-specific self-antigen are qualitatively more suppressive than non-specific polyclonal Tregs (31–34). The problem is that Tregs of a particular antigen-specificity are present in very limited numbers in peripheral blood (33–35). The solution requires innovative strategies that yield substantial antigen-specific Treg populations via continuous expansion in long-term cultures. To devise novel Treg-based *in vitro* expansion strategies, we explored the concept that recombinant mouse proteins comprised of fused CD25 and IL-2 domains would serve as partial agonists for the IL-2 receptor and would provide a broad concentration window that favored Treg outgrowth and dominance in continuous expansion cultures. These CD25-IL2 and IL2-CD25 FPs were substantially less active than IL-2, exhibited transient binding to transmembrane cell-surface CD25, and exhibited broad bell-shaped concentration ranges that favored virtually complete Treg dominance during continuous culture (∼98% Treg purity). When tested *in vitro*, FP-induced Tregs suppressed antigen-specific proliferation of naïve CD4^+^ responder T cells and promoted FOXP3 expression in a responder T cell subset. When administered *in vivo*, these FPs elicited robust increases in circulating Tregs and strongly augmented CD25 expression on Tregs but not on Tcons by antigen-independent mechanisms. Notably, tolerogenic vaccination coupled with subsequent boosting with these FPs maintained CD25^high^ Tregs throughout a 30-day observation window. Overall, these data are consistent with the concept that CD25-IL2 and IL2-CD25 FPs impose qualitative limitations in IL-2 signaling that favor dominance of FOXP3^+^ CD25^high^ Tregs *in vitro* and *in vivo*. A similar study published during the course of this study also revealed that IL2-CD25 FPs had selective growth potential for Tregs (36).

## Materials and Methods

### Mice

C57BL/6 mice (Stock Number 000664), CD45.1 mice (B6.SJL-Ptprca Pepcb/BoyJ Stock Number 002014), Foxp3-IRES-GFP (FIG) mice (B6.Cg-Foxp3^tm2Tch^/J Stock Number 006772), MOG35-55 specific TCR transgenic 2D2 mice (C57BL/6-Tg(Tcra2D2,Tcrb2D2)1Kuch/J Stock Number 006912), and OVA323-339 specific TCR transgenic OTII mice (B6.Cg-Tg(TcraTcrb)425Cbn/J Stock Number 004194) were obtained from Jackson Laboratory (Bar Harbor, ME, United States) and maintained as a colony in the Department of Comparative Medicine at East Carolina University. CD45.1 2D2-FIG, 2D2-FIG, and OTII-FIG mouse strains were derived through intercross breeding and routinely screened by FACS analysis of peripheral blood mononuclear cells (PBMCs) with antibodies for the CD45.1/CD45.2 alleles, the 2D2 Vβ11 and Vα3.2 TCR proteins, and the OTII Vβ5.1/5.2 and Vα2 TCR proteins. GFP expression from FIG mice was used as a surrogate marker of FOXP3 expression. Animal care and use was performed in accordance with approved animal use protocols and guidelines of the East Carolina University Institutional Animal Care and Use Committee.

### Generation and Purification of Recombinant Fusion Proteins

The FP IL2-CD25 included murine IL-2 (accession number NP_032392) as the N-terminus, a linker, murine soluble CD25 (accession number NP_032393), and a C-terminal poly-histidine affinity purification tag. The sequence was as follows: (IL-2 aa1-169)-G-G-G-G-S-T-R-G-G-G-G-S-T-G-G-G-G-S-G-G-G-G-S-(soluble CD25 aa22-236)-H-H-H-H-H-H-H-H. The FP CD25-IL2 included murine soluble CD25 as the N-terminus, a linker, murine IL-2, and a C-terminal 8-histidine affinity purification tag. The sequence was as follows: (soluble CD25 aa1-236)-G-G-G-G-S-T-R-G-G-G-G-S-T-G-G-G-G-S-G-G-G-G-S-(IL-2 aa20-169)-H-H-H-H-H-H-H-H. Full length murine CD25 included the full aa1-268 sequence. Murine soluble CD25 included the N-terminal aa1-208 soluble domain with a C-terminal 8-histidine tag. Genes encoding IL2-CD25, full length CD25, and sCD25 were cloned into the pIRES AcGFP1 expression vector (Clontech). These three expression vectors were used to stably transfect HEK293F cells, and the surrogate GFP marker was used to detect transfected cells. The CD25-IL2 gene sequence was cloned into the pCMV expression vector and used to stably transfect HEK293F cells. Expression supernatants were concentrated on YM10 ultrafiltration membranes and then directly loaded onto Ni-NTA resin followed by extensive washing (50 mM NaH_2_PO_4_, 500 mM NaCl, with 20, 40, or 60 mM imidazole, pH 8.0). Recombinant FPs were eluted with 250 mM imidazole (pH 8.0) and were concentrated and diafiltrated against phosphate buffered saline in Amicon Ultra-15 centrifugal filters (EMD Millipore, Billerica, MA, United States). Protein quantity was assessed by absorbance at 280 nm, and purity was assessed by SDS-PAGE. Bioactivities of IL2-CD25 and CD25-IL2 were validated by proliferation of an IL-2-dependent T cell line (i.e., the IL-2 dependent transformed SJL-PLP.1 T cell line). Bioactivity of sCD25 was validated by blocking the IL-2-stimulated proliferation of the SJL-PLP.1 T cell line. Expression of full length CD25 was validated by flow cytometric analysis.

### Synthetic Peptides, TGF-β, GMCSF-MOG, and IL-2

Synthetic peptides MOG35-55 (MEVGWYRSPFSRVVH LYRNGK) and OVA323-339 (ISQAVHAAHAEINEAGR) were obtained from Genscript (Piscataway, NJ, United States). Recombinant rat TGF-β1 was expressed by transfected HEK293F cells. TGF-β1 was expressed and purified as described in previous studies (30, 37). The purified protein was activated by 10 min of exposure to 70°C, and each prep was verified for bioactivity by induction of FOXP3 in MOG-stimulated 2D2-FIG splenocyte (SPL) cultures. Generation, expression, purification, and bioassay of GMCSF-MOG was performed as described in previous studies (16, 19, 20). This FP was comprised of the murine GM-CSF cytokine as the N-terminal domain, the peptide sequence comprising MOG35-55, and an eight-histidine C-terminus. GMCSF-MOG was expressed from transfected, stable lines of HEK and CHO (Chinese hamster ovary) cells. Recombinant murine IL-2 (accession number NP_032392) was purified from a transfected stable HEK293F cell line. Bioactivity of purified IL-2 protein was assessed by proliferation of the IL-2-dependent SJL-PLP.1 T cell line. Protein quantity was assessed by absorbance at 280 nm, and purity was assessed by SDS-PAGE.

### Generation and Purification of the Anti-CD25 PC61 mAb

The hybridoma secreting the anti-mouse CD25 PC61-5.3 mAb was cultured in supplemented DMEM in C2011 hollow fiber cartridges (FiberCell Systems, Frederick, MD, United States) as described in a previous study (38). Supernatants were cleared at 7,200 × *g*, precipitated with 50% ammonium sulfate, and dissolved in phosphate buffered saline. The mAb preparations were purified via protein G agarose columns and eluted with 200 mM glycine at pH 3.0 and immediately neutralized by 1 M Tris buffer at pH 9.0. Antibody purity was assessed by SDS-PAGE. PC61 activity was assessed by blocking IL-2 dependent T cell responses and by flow cytometric assays.

### Flow Cytometric Analyses of PBMCs and Lymphocytes

Blood from the submandibular vein was collected into 200 μl of sodium citrate (130 mM). Cells were washed in 3 ml HBSS and stained for 1 h at 4°C in the dark with designated cocktails of fluorochrome-conjugated antibodies. After staining, red blood cells were lysed by incubation for 10 min on ice with 3 ml of ammonium chloride lysis buffer (150 mM NH_4_Cl, 10 mM NaHCO_3_, and 1.2 mM EDTA pH 7.2). Samples were washed three times with HBSS and analyzed by use of a Becton-Dickson LSRII flow cytometer (San Jose, CA, United States) followed by analysis with FlowJo software (Ashland, OR, United States). In designated experiments, reference beads (AccuCount PE- or APC-conjugated EasyComp fluorescent particles 3.0–3.4 μm, Spherotech, Lake Forest, IL, United States) were added to samples immediately before cytometric analysis to assess absolute cell numbers. Fluorochrome-conjugated mAbs were obtained from BioLegend and were specific for CD3 (17A2 or 145-2C11), CD4 (GK1.5), CD25 (PC61, 7D4, and 3C7), CD45.1 (A20), CD45.2 (104), CD69 (H1.2F3), Histag (J095G46), Neuropilin-1 (NRP-1, 3E12), TCR Vβ11 (KT11), TCR Vα3.2 (RR3-16), TCR Vβ5.1,5.2 (MR9-4), and TCR Vα2 (B20.1).

### Generation and Cultivation of Treg Lines

Naïve SPL were harvested from 2D2-FIG or OTII-FIG mice and were activated with 1 μM MOG35-55 or 100 nM OVA323-339, respectively, at a density of 2 × 10^6^/ml in complete RPMI (10% heat-inactivated fetal bovine serum, 2 mM glutamine, 100 μg/ml streptomycin, 100 U/ml penicillin, 50 μM 2-ME) for 3–4 days in the presence of 10 nM TGF-β to elicit Treg differentiation. After the initial activation, T cells were propagated and passaged every 3–4 days in complete RPMI containing IL2-CD25, CD25-IL2, or IL-2 with or without the anti-CD25 PC61 mAb as designated (30). Periodically, Tregs were reactivated at a density of 1 × 10^6^/ml in complete RPMI in the presence of IL-2 for 3–4 days with irradiated dendritic cells (DCs) (1:10 DC:Treg ratio), 1 μM MOG35-55 or 100 nM OVA323-339, and 10 nM TGF-β.

### Size Exclusion Chromatography

Size exclusion chromatography experiments were conducted on an ÄKTA pure 25L FPLC (GE Healthcare) and a Superdex 200 Increase 10/300 GL column (GE Healthcare) with a flow rate of 0.5 ml/min in HEPES-buffered saline (10 mM HEPES pH 7.3, 140 mM NaCl). A sample volume of 500 μl (0.5 mg/ml) was run on the column. Relative molecular weights were determined via a semi-log linear fit relative to a Bio-Rad Gel Filtration Standard, which included globular proteins γ-globulin, ovalbumin, myoglobin, and vitamin B12.

### *In vitro* Suppression Assays

#### Treg-Mediated Suppression via Responder T Cell Proliferation

T cell responders were isolated from a naïve 2D2-FIG mouse by use of a CD4^+^ MACS system (Miltenyi Biotec, Bergisch Gladbach, Germany). As designated, CD45.2 2D2-FIG Tregs were cultured with purified naive 2D2-FIG responder cells (25,000/well) in the presence of irradiated C57BL/6 splenocytes (50,000/well) with or without 1 μM MOG^35–55^ in complete RPMI for 3 days. Cultures were pulsed with 1 μCi [^3^H]thymidine (6.7 Ci/mmol, New England Nuclear, Perkin Elmer, Waltham, MA, United States) during the last 24 h of a 72-h culture. Cultures were then harvested onto filters by use of a Tomtec Mach III harvester (Hamden, CT, United States). [^3^H]thymidine incorporation into DNA was measured by use of a Perkin Elmer MicroBeta2 liquid scintillation counter.

#### Treg-Mediated Suppression via Responder Phenotype

Treg-mediated suppression of responder T cells was assessed by flow cytometric analysis. T cell responders were isolated from a CD45.1 2D2-FIG mouse and purified via a CD4^+^ MACS system. CD45.2 2D2-FIG Tregs from a continuous line were cultured with purified CD45.1 2D2-FIG responder cells (50,000/well) and activated with irradiated C57BL/6 DCs (10,000/well) with or without 1 μM MOG^35–55^ in complete RPMI. After 3 days, cells were stained, washed, and analyzed by use of a Becton-Dickson LSRII flow cytometer.

### Induction and Assessment of EAE

Complete Freund’s Adjuvant (CFA) (Incomplete Freund’s Adjuvant with 4 mg/ml heat-killed *Mycobacterium tuberculosis* H37Ra, BD Biosciences, Franklin Lakes, NJ, United States) was mixed 1:1 with MOG35-55 in phosphate-buffered saline and then was emulsified by sonication. To induce EAE, C57BL/6 mice were injected with 200 μg MOG35-55 in a total volume of 100 μl emulsion via three subcutaneous injections of 33 μl across the lower back. Each mouse received separate intraperitoneal injections (400 ng) of Pertussis toxin on days 0 and 2. All immunizations were performed under isoflurane anesthesia (Abbott Laboratories, Chicago, IL, United States). Mice were assessed daily for clinical score based on an algorithm commonly used in the field (39). The following scale was used to score the clinical signs of EAE: 0, no disease; 0.5, partial paralysis of tail without ataxia; 1.0, flaccid paralysis of tail or ataxia but not both; 2.0, flaccid paralysis of tail with ataxia or impaired righting reflex; 3.0, partial hind limb paralysis marked by inability to walk upright but with ambulatory rhythm in both legs; 3.5, same as above but with full paralysis of one leg; 4.0, full hindlimb paralysis; 5.0, total hindlimb paralysis with forelimb involvement or moribund. A score of 5.0 was a humane endpoint for euthanasia. Mice that did not exhibit EAE had a score of zero, and these scores were included in the group average. Mice that exhibited humane endpoints as assessed by weight loss or clinical score of 5.0 were subjected to humane euthanasia and were omitted from scoring thereafter. Time-course graphs portrayed daily mean clinical scores. Mice were also assessed daily for body weight. To calculate percent weight loss, 100% body weight was assigned as the maximal body weight during days 0–10, and daily body weights were calculated for each day after normalization to this 100% value. Mean EAE clinical scores and percent initial body weight data were shown with the standard error of the mean (SEM).

### Preparation of Vaccines

Immunizations containing CD25-IL2 or IL2-CD25 (2–3 nmoles as designated) or GMCSF-MOG (4 nmoles) were administered subcutaneously in saline or Alum. CD25-IL2/Alum-based vaccines were prepared by mixing equal volumes of Alhydrogel adjuvant (InvivoGen) and CD25-IL2 (in saline) for an injection volume of 100 μl per mouse. The Alum/vaccine mixture was incubated for 1 h on ice with continuous agitation to allow the protein to attach to the Alum gel. The vaccine was administered by two subcutaneous injections of 50 μl each. All immunizations were performed under isoflurane anesthesia (Abbott Laboratories, Chicago, IL, United States).

### Statistical Analysis

To determine statistical significance, comparisons among three groups or more were analyzed via use of ANOVA with the Holm-Sidak multiple comparisons test. Comparisons between two groups were analyzed by Student’s *t*-test. A *p*-value < 0.05 was considered significant.

## Results

### CD25-IL2 and IL2-CD25 Were Constrained Partial IL-2 Agonists

**FIGURE 1:**
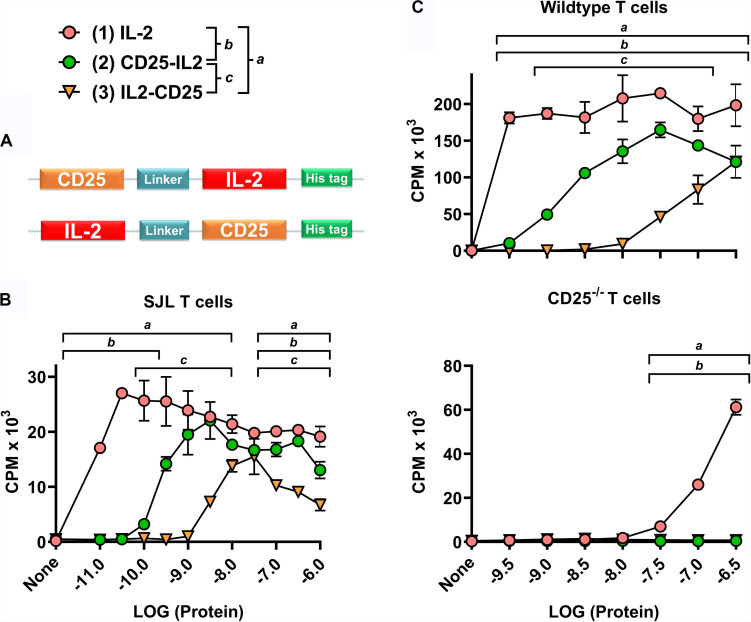
CD25-IL2 and IL2-CD25 were partial IL-2 agonists. **(A)** N-terminal CD25 or IL-2 domains were fused via a 23-aa flexible linker with C-terminal IL-2 or CD25 domains, respectively, to generate CD25-IL2 and IL2-CD25 FPs. These FPs were comprised of murine sequences. **(B)** A transformed, IL-2 dependent mouse T cell line (SJL-PLP.1) was cultured at 5,000 cells/well with designated concentrations of purified mouse IL-2, CD25-IL2, or IL2-CD25. **(C)** Wild type (top) or CD25^–/–^ (bottom) SPLs were activated at a density of 2 × 10^6^ cells/ml with 2.5 μg/ml concanavalin A. After 3 days of activation, CD4^+^ blastogenic T cells were purified and cultured at 25,000 cells/well with designated concentrations of purified proteins. **(B,C)** Cultures were pulsed with [^3^H]thymidine during the last 24 h of a 3-day culture and were harvested to measure cellular proliferation. The *y*-axes represent counts per minute (CPM), and error bars represent the standard deviation (SD). Note use of different *y*-axis scales. Statistical significance was analyzed by use of a one-way ANOVA with the Holm-Sidak multiple comparisons test. Statistically significant (*p* < 0.05) differences were noted as indicated (*a*, 1 vs 3) (*b*, 1 vs 2) (c, 2 vs 3). These data represent three independent experiments.

CD25-IL2 and IL2-CD25 FPs (Figure 1A) were devised to lock IL-2 signaling into a constrained low-zone IL-2 signaling mode. We hypothesized that these FPs would sequester the FP-IL2 domain in an occluded reservoir of *cis* or *trans* conformers to provide an equilibrium-based, steady-state supply of the accessible bioactive FP-IL2 domain. According to this model, the bioactive accessible IL2-FP domain would be generated continually at low concentrations to provide low-zone IL-2 signaling via transmembrane IL2Rαβγ to establish dominant outgrowth of FOXP3^+^ Tregs. As such, these FPs may represent suitable tools to test the concept that low-zone IL-2 signaling defines a key dimension of the ‘Treg niche.’

Both CD25-IL2 and IL2-CD25 exhibited IL-2 bioactivity, although the respective FPs were approximately 32- and 320-fold less potent than mouse IL-2 in assays measuring proliferation of a transformed IL-2 dependent mouse T cell line (SJL-PLP.1 T cells) (Figure 1B). Overall, these data confirmed the prediction that the engineered recombinant FPs represented a low-zone IL-2 signaling modality. An important question was whether the FPs directly engaged and signaled through the low-affinity CD122/CD132 IL2R-βγ heterodimer. The alternative possibility was that the FPs exhibited an equilibrium in which active accessible conformations were in flux with inactive inaccessible conformations, which, respectively, would or would not provide an accessible FP-IL2 domain free to interact with the high-affinity CD25/CD122/CD132 transmembrane IL2R-αβγ trimer. To address this issue, wild type or CD25^–/–^ SPLs were activated with 2.5 μg/ml concanavalin-A for 3 days, and the resulting blastogenic CD4^+^ T cells were purified to obtain CD25^high^ or CD25^–/–^ blastogenic responders. The rank order of activity for CD25^high^ wild type T cells was (IL-2 > > CD25-IL2 > IL2-CD25) (Figure 1C, top). As compared to CD25^high^ wild type T cells, IL-2 was at least 1000-fold less active in stimulating CD25^–/–^ blast T cells, and the FPs had no activity (Figure 1C, bottom). These findings underline the critical role of transmembrane CD25 in mediating IL-2 and FP-IL2 responsiveness of T cells. If a higher-order FP conformer (e.g., a cis CD25-IL2 hairpin or an anti-parallel CD25-IL2 homodimer) directly mediated trans-IL-2 signaling via a IL2R-βγ receptor, one would predict that the FPs would be more potent than IL-2 in assays of CD25^–/–^ T cells simply because the FP-CD25 domain should contribute affinity to the interaction with transmembrane CD122/CD132. But this prediction was not the observed outcome. Rather, the data are most consistent with the possibility that an unencumbered FP-IL2 domain in an active accessible FP conformation engaged the intact transmembrane IL2R-αβγ receptor. The signaling requirement for transmembrane CD25 suggested that these FPs can be exploited to selectively target the low-zone IL-2 Treg niche to drive dominant Treg responses.

### Fusion Proteins Exhibited Transient Binding to Cell-Surface Transmembrane CD25

**FIGURE 2:**
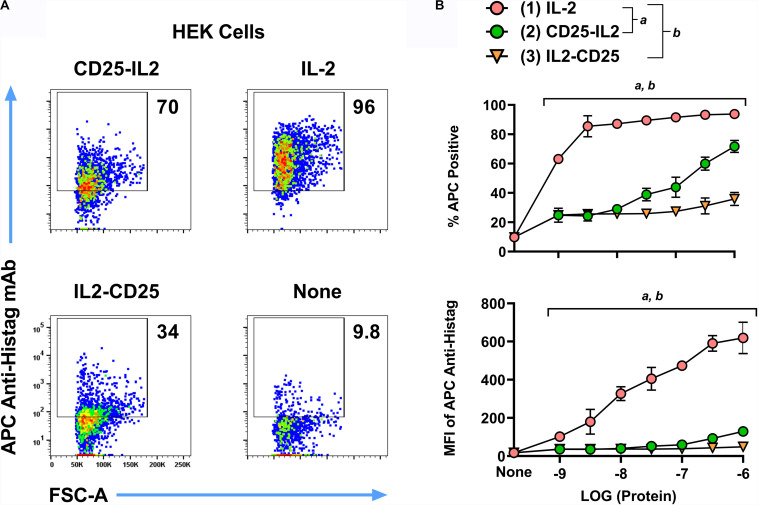
Fusion proteins exhibited transient binding to cell-surface transmembrane CD25. HEK cells expressing transmembrane full-length mouse CD25 were incubated with 1 μM **(A)** or designated concentrations (**B**, *x*-axis) of the His-tagged proteins CD25-IL2, IL2-CD25, or IL-2. These cells were extensively washed and were incubated with an APC-labeled anti-Histag mAb. Shown are **(A)** representative dotplots together with percentages of APC^+^ cells (**B**, top) and the anti-Histag MFI of GFP^+^ cells (**B**, bottom). Statistical significance was analyzed by use of a one-way ANOVA with the Holm-Sidak multiple comparisons test. Statistically significant (*p* < 0.05) differences were noted as indicated (*a*, 1 vs 2) (*b*, 1 vs 3). Error bars represent SD. These data represent three independent experiments.

To test the model that the CD25-IL2 and IL2-CD25 FPs exhibited an equilibrium between inactive inaccessible conformations vs active accessible conformations featuring an unencumbered FP-IL2 domain, we tested whether the FPs directly bound HEK cells that expressed transmembrane full-length CD25 (HEK-fl-CD25) in the absence of IL2R-βγ. The prediction is that the inaccessible FP conformer would exhibit a sequestered IL-2 domain and thereby would lack binding to transmembrane CD25. Conversely, the accessible FP conformer would have an unencumbered IL-2 domain that would bind transmembrane CD25. To assess this issue, we incubated HEK-fl-CD25 cells with designated concentrations of the His-tagged CD25-IL2, IL2-CD25, or IL-2 recombinant proteins. The cells were washed and then incubated with an APC-labeled anti-Histag mAb. At high concentrations (1 μM), CD25-IL2, IL2-CD25, and IL-2 proteins differentially labeled HEK-fl-CD25 cells with a rank order of IL-2 > > CD25-IL2 > IL2-CD25 (Figure 2A). The CD25-IL2 and IL2-CD25 FPs exhibited progressively less binding or no appreciable binding activity at lower concentration ranges as measured by percentages of APC^+^ HEK-fl-CD25^+^ cells or by mean fluorescence intensity (APC MFI) (Figure 2B). The rank order of binding activity paralleled the rank order of proliferative activity noted in Figures 1B,C. These data support the equilibrium model postulating accessible and inaccessible FP conformations, with the accessible FP-IL2 conformation representing the active form.

### The IL-2 Binding Site of CD25 Was Occluded in the CD25-IL2 and IL2-CD25 FPs

**FIGURE 3:**
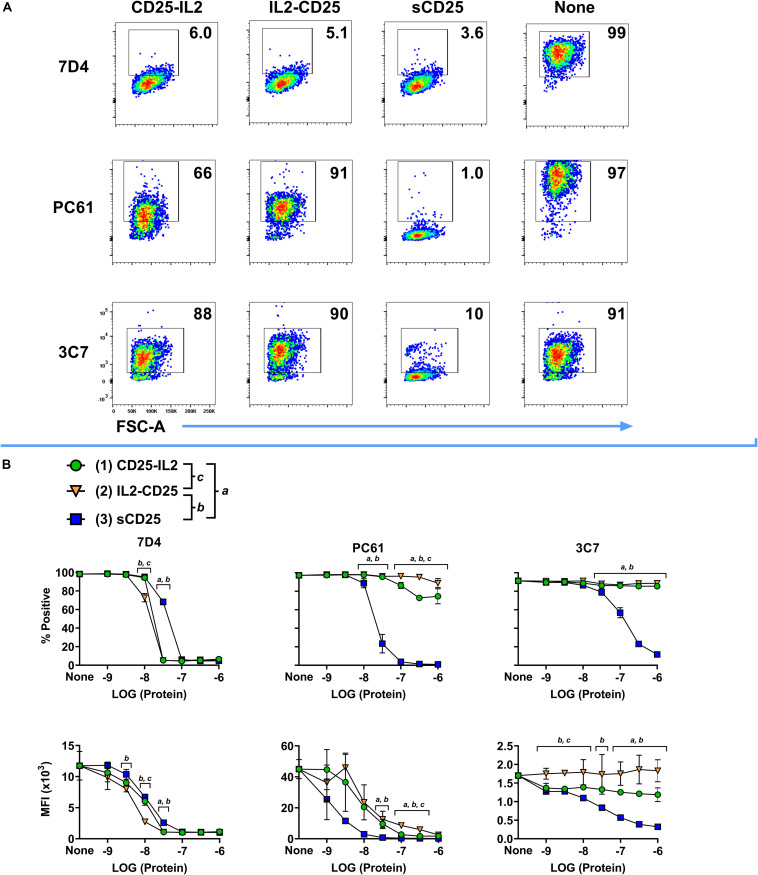
The IL-2 binding site of CD25 was occluded in the CD25-IL2 and IL2-CD25 FPs. The fluorochrome-labeled anti-CD25 mAbs PE-3C7, APC-PC61, or BV421-7D4 were incubated with or without designated concentrations of CD25-IL2, IL2-CD25, or soluble CD25 followed by incubation with 200,000 HEK cells that expressed full length transmembrane mouse CD25. Shown are **(A)** representative dot plots after designated anti-CD25 mAbs were incubated with 1 μM of designated proteins and **(B)** percentages (top row) and MFI values (bottom row) of GFP^+^ HEK cells after the designated anti-CD25 mAbs were incubated with designated concentrations of the recombinant proteins. Statistical significance was analyzed by use of a one-way ANOVA with the Holm-Sidak multiple comparisons test. Statistically significant (*p* < 0.05) differences were noted as indicated (*a*, 1 vs 3) (*b*, 2 vs 3) (*c*, 1 vs 2). Error bars represent SD. These data represent three independent experiments.

According to the active accessible vs inactive inaccessible model of FP conformers, the IL-2 binding pocket of CD25 should be occluded in the inactive conformers but should be accessible in the active conformer. To test for the potential occlusion of the IL-2 binding site of the CD25 domain, fluorochrome-labeled mAbs specific for distinct epitopes of CD25 were incubated with varying concentrations of CD25-IL2, IL2-CD25, or soluble CD25 (sCD25) followed by incubation with HEK-fl-CD25^+^ cells. The fluorochrome-labeled anti-mouse CD25 mAb included two mAbs (7D4 and PC61) that bound non-competitive epitopes outside the IL-2 binding site of CD25 and one mAb (3C7) known to directly occlude the IL-2 binding pocket of CD25 (40–42). In regard to interactions of the BV421-7D4 mAb with HEK-fl-CD25^+^ cells, high concentrations of CD25-IL2, IL2-CD25, and sCD25 intercepted the 7D4 mAb and prevented binding of the mAb to CD25^+^ HEKs (Figure 3A). The FPs neutralized the 7D4 mAb in a concentration-dependent manner as measured by percentages of mAb-labeled HEK-fl-CD25^+^ cells or by MFIs (Figure 3B). Notably, both FPs were more potent than sCD25 in blocking the 7D4 mAb, which provided suggestive evidence that the interactions between the FP-IL2 and FP-CD25 domains enhanced availability of the 7D4-specific epitope on the FP-CD25 domain. In regard to interactions of the APC-PC61 mAb with HEK-fl-CD25^+^ cells, high CD25-IL2 and IL2-CD25 concentrations were only partially effective in blocking the PC61 mAb whereas sCD25 fully blocked binding of the mAb to HEK-fl-CD25^+^ cells (Figure 3A). Lower concentration ranges of the FPs also were substantially less effective compared to sCD25 in blocking the PC61 mAb (Figure 3B). These data indicated that FP-IL2 domain robustly blocked the availability of a PC61-specific epitope on the FP-CD25 domain. This finding is consistent with published evidence of non-competitive allosteric interactions between IL-2 and PC61 in binding CD25 (42). CD25-IL2 was more effective than IL2-CD25 for neutralization of the PC61 mAb, which provided evidence that the PC61-specific binding site was more disrupted in IL2-CD25 compared to CD25-IL2. In regard to interactions of the PE-3C7 mAb with HEK-fl-CD25^+^ cells, the 3C7 epitope on CD25 was fully sequestered in CD25-IL2 and IL2-CD25 but was freely available in sCD25 (Figures 3A,B). These findings implied that CD25-IL2 and IL2-CD25 FPs formed structures that occluded the 3C7/IL-2 binding site of CD25, whereas CD25 epitopes for PC61 and 7D4 mAbs were partially or fully available, respectively. Overall, these data provided additional evidence that the FPs mediated low-efficiency IL-2 signaling due to an equilibrium between free and occluded FP-IL2 domains with kinetic dominance by inactive complexes in which the FP-CD25 domain bound the FP-IL2 domain to hinder IL-2 bioactivity.

### CD25-IL2 and IL2-CD25 FPs Formed Higher Order Multimers

**FIGURE 4:**
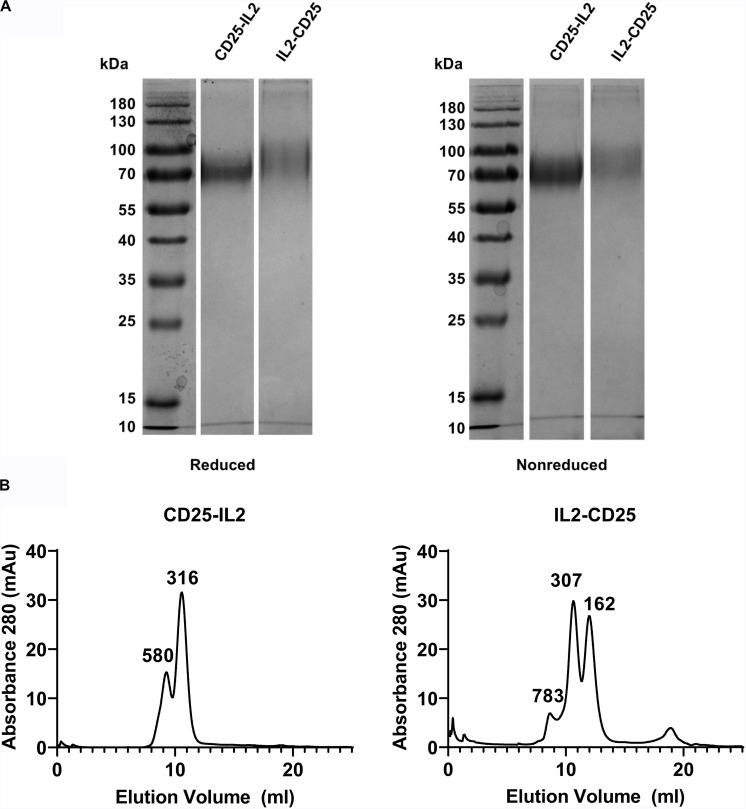
CD25-IL2 and IL2-CD25 FPs formed higher-order multimers. **(A)** Shown are SDS-PAGE gels of purified FPs under reducing and non-reducing conditions. Proteins were identified by the InstantBlue Protein stain. **(B)** The indicated FPs were subjected to size exclusion chromatography by use of an ÄKTA pure 25L FPLC and a Superdex^TM^ 200 Increase 10/300 GL column. The elution profiles are shown with apparent molecular weights (kDa) for each peak as indicated.

Physical characterization also supported the hypothesis that these FPs exhibited non-covalent intramolecular and/or intermolecular bonding to form higher MW oligomers. First, in that both the IL-2 and CD25 domains of each FP have an unpaired cysteine residue, we tested whether the predicted oligomers were stabilized by disulfide linkages. SDS-PAGE under reducing and non-reducing conditions revealed dispersed bands at ∼70 kDa consistent with a glycosylated monomeric form of both FPs (Figure 4A). These data discounted the possibility that multimer formation was due to *trans* disulfide linkages between FP domains. Second, to understand if the FPs formed higher order oligomers in solution, size exclusion chromatography was performed on both purified FPs. Analysis of the apparent molecular weights revealed higher order multimers for both FPs without evidence of a major monomeric species (Figure 4B). The analysis supported major protein species of 316 kDa and 307 kDa for CD25-IL2 and IL2-CD25, respectively, which were inconsistent with the expected monomer mass. These data suggest that both FPs lack a major monomeric species in solution. The hypothesis of a flux among active and inactive conformations was supported by the presence of multiple apparent molecular weights for each FP indicating an equilibrium of multiple molecular forms comprising higher order multimers. However, size exclusion chromatography cannot distinguish among numerous possibilities of oligomeric structure including hairpin formation by *cis* and/or *trans* ligand-receptor interactions. Non-covalent reversible interactions among multiple FP molecules would allow for the equilibrated presence of a conformer with an accessible active IL-2 domain. If so, then the major species of occluded FP multimers would be a reservoir providing a steady-state low-zone concentration of exposed FP-IL2 signaling domains that would bind transmembrane high-affinity CD25/CD122/CD132 IL2R-αβγ trimer on T cells, leading to IL-2 signaling.

### Low Concentrations of CD25-IL2 and IL2-CD25 FPs Selectively Favored Treg Dominance *in vitro*

The utility of understanding the active and inactive multimer FP conformers is that direct signaling of the CD25-IL2 complex via the IL2Rβγ receptor would not predictably favor Tregs, because Tregs and Tcons express comparable amounts of IL2Rβγ. Conversely, direct signaling of a free, accessible FP-IL2 domain in an active conformer would predictably favor Tregs, because Tregs express substantially higher surface CD25 levels compared to Tcons. Thus, the utility and underlying mechanism of these FPs can be tested by assessing whether these FPs facilitate dominant outgrowth of Tregs in continuous culture systems.

**FIGURE 5:**
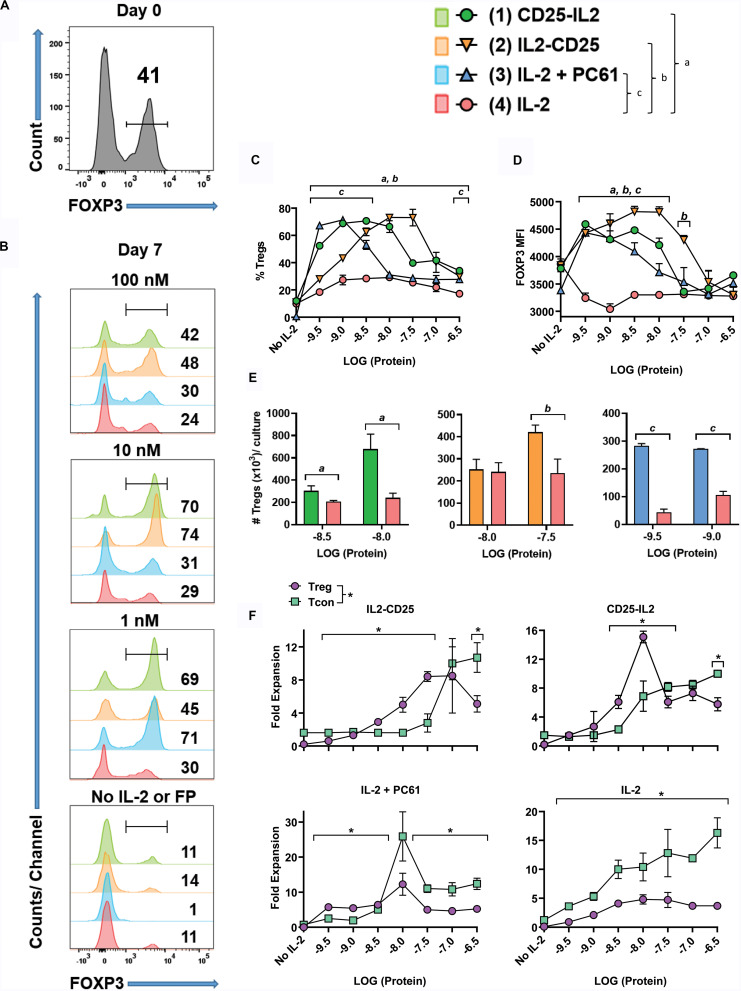
Low concentrations of CD25-IL2 and IL2-CD25 FPs selectively favored Treg dominance *in vitro*. OTII-FIG SPLs were activated at a density of 2 × 10^6^ cells/ml in complete RPMI medium with 100 nM OVA323-339 and 10 nM TGF-β. **(A)** After 3 days of activation (designated day 0), cells were analyzed for FOXP3 expression. **(B–F)** The mixed Treg/Tcon line (approximately 40/60%, respectively) was cultured at 1 × 10^5^/200 μl/well in triplicate cultures with designated concentrations of CD25-IL2, IL2-CD25, IL-2 + PC61, or IL-2. In the “IL-2 + PC61” group, designated concentrations of IL-2 were included with a standard 10 μg/ml concentration of the anti-CD25 PC61 mAb. On day 7, cells were analyzed for FOXP3 expression. Shown are **(B)** histograms of FOXP3 expression at designated concentrations, **(C)** percentages of FOXP3^+^ Tregs among the CD4 ^+^T cell population, **(D)** the FOXP3 MFI among the Treg population, **(E)** total number of FOXP3^+^ Tregs per culture, and **(F)** the fold expansion of both the FOXP3^+^ Treg and FOXP3^–^ Tcon population per culture. Error bars represent SD. **(C–E)** Statistical significance was analyzed by use of a one-way ANOVA with the Holm-Sidak multiple comparisons test comparing each group mean to the group 4 (IL-2) mean. Statistically significant (*p* < 0.05) differences were noted as indicated (*a*, 1 vs 4) (*b*, 2 vs 4) (c, 3 vs 4). **(F)** Statistical significance was analyzed by use of *t*-test comparing the Treg mean to the Tcon mean for each concentration (*p < 0.05). These data represent three independent experiments.

To address whether the FPs enable competitive outgrowth of Tregs, OTII-FIG SPLs were activated for 3 days with 100 nM ovalbumin 323-339 (OVA323-339) and 10 nM TGF-β to generate a mixed Treg/Tcon line (approximately 40/60%, respectively) (Figure 5A). The Treg population most likely was comprised of expanded populations of natural Tregs together with TGF-β-induced Tregs that derived from naïve T cell precursors. Designated concentrations (Figures 5B–F) of CD25-IL2, IL2-CD25, IL-2 plus 10 μg/ml PC61, or IL-2 alone were used to propagate this line during an additional 7-day culture. The combination of IL-2 plus PC61 was used as a positive control because previous studies showed that these conditions promoted Treg outgrowth (30). As expected, low IL-2 concentrations (1 nM) coupled with high PC61 concentrations resulted in stabilization and outgrowth of Tregs to 71% (Figure 5B). The FPs, when added at concentrations of 1 and 10 nM, elicited percentages of FOXP3^+^ Tregs (CD25-IL2: 69 and 70%; IL2-CD25: 45 and 74%, respectively) that were significantly higher than the percentage of Tregs stabilized by IL-2 alone (∼29–30%, Figure 5B). The Treg-stabilizing actions of these FPs reflected bell-shaped concentration-response curves, because higher concentrations of FPs (100 nM) resulted in lower Treg percentages (Figures 5B,C). As noted for the rank order of potency (IL-2 > > CD25-IL2 > IL2-CD25) in IL-2 proliferative assays and cell binding assays (Figures 1, 2), the bell-shaped curve for CD25-IL2 was left-shifted compared to that for IL2-CD25 (Figure 5C). Thus, lower concentrations of CD25-IL2 were required to establish adequate low-zone IL-2 concentrations needed for Treg dominance compared to IL2-CD25. These data provide evidence that the FP-IL2 domain was more tightly buried in the IL2-CD25 FP. The Treg-favorable actions of the FPs within the defined low-zone IL-2 window not only enabled Treg dominance but also facilitated increased FOXP3 expression as assessed by the MFI (Figure 5D). Both CD25-IL2 and IL2-CD25 FPs enabled the selective expansion of Tregs within the respective low-zone IL-2 window as assessed by Treg numbers (Figure 5E). The hypothesis that low-zone vs high-zone IL-2 signaling ranges, respectively, controlled dominance of Tregs and Tcons was supported by assessments of Treg and Tcon yield as a function of FP concentration (Figure 5F). Low concentrations (3.2–32 nM IL2-CD25; 1.0–10 nM CD25-IL2; 320 fM – 1 nM IL2 in the presence of PC61) favored higher yields of Tregs whereas higher concentrations of each modality favored higher yields of Tcons. Notably, IL-2 did not establish a favorable Treg-zone at any concentration. These data indicate that a low IL-2 signaling zone favorable to Tregs cannot be established by IL-2 alone. Rather, IL-2 requires a buffering modality to maintain consistent low concentrations of IL-2 over time to promote Treg dominance.

### CD25-IL2 and IL2-CD25 FPs Enabled Dominant Outgrowth of FOXP3^+^ Treg Lines During Continuous Culture

**FIGURE 6:**
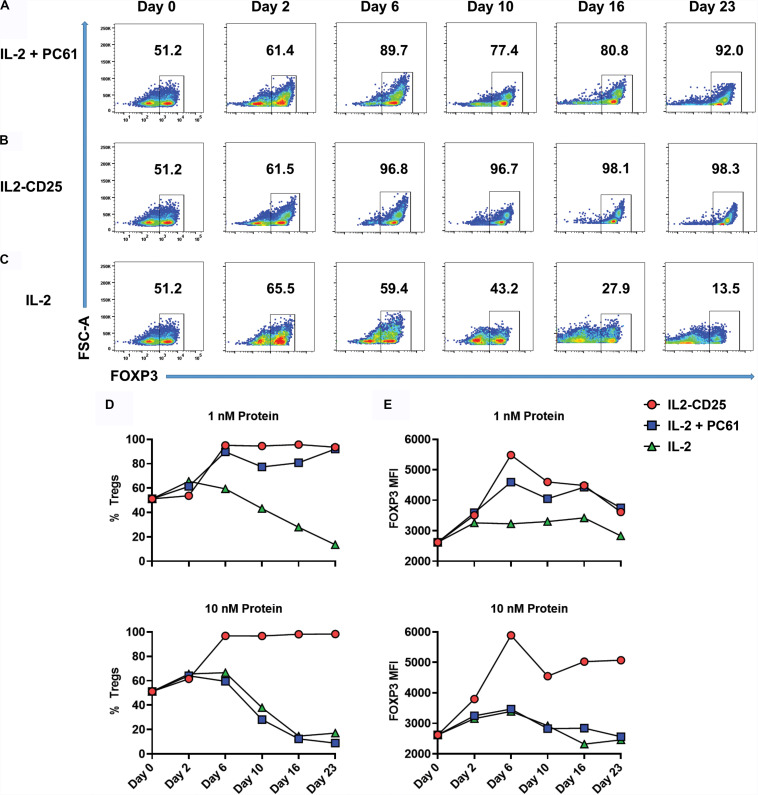
IL2-CD25 enabled dominance of FOXP3^+^ Treg lines during continuous culture. 2D2-FIG SPLs were activated at a density of 2 × 10^6^ cells/ml in complete RPMI medium with 1 μM MOG35-55 and 10 nM TGF-β. After 3 days of activation, cells were passaged (designed day 0) at a density of 10^6^ cells/ml with 1 nM mouse IL-2 and 10 μg/ml PC61 **(A)**, 10 nM IL2-CD25 **(B)**, or 1 nM mouse IL-2 **(C)**. Cells were passaged in these conditions every 3–4 days and were analyzed for percentages of CD4^+^ FOXP3^+^ T cells on designated days. Shown are the percentages of CD4^+^ FOXP3^+^ Tregs **(D)** and the FOXP3 MFI (CD4^+^ FOXP3^+^ gate) **(E)** when propagated with 1 nM (top) or 10 nM (bottom) of IL-2 or IL2-CD25 (PC61 = 10 μg/ml). These data represent three independent experiments.

Because CD25-IL2 and IL2-CD25 favored Treg dominance within defined low concentration windows over a 7-day period (Figure 5), a central question was whether continuous propagation of mixed Treg/Tcon lines in these FPs culminated in Treg monocultures. To address this question, Tregs were generated by culturing 2D2-FIG splenocytes with 1 μM MOG35-55 and 10 nM TGF-β in a 3-day activation to produce a line of ∼50% Tregs. When passaged out of this initial activation culture (day 0), T cells were cultured with 1 or 10 nM of IL2-CD25, IL-2 alone, or IL-2 plus 10 μg/ml of PC61 mAb every 3–4 days for 23 days (Figures 6A–C). Cultures supplemented with 1 nM IL-2/PC61 exhibited high Treg percentages from days 6–23 (Figure 6A). In cultures supplemented with 1 or 10 nM IL2-CD25, CD4^+^ FOXP3^+^ Tregs represented over 90% of the T cells by day 6, and these levels were maintained throughout the duration of the time course (Figures 6B,D). Conversely, T cell lines propagated in 1 nM IL-2 showed a steady loss of Tregs with only 13.5% Tregs remaining at day 23, and T cells cultured in 10 nM IL-2 were likewise overgrown by Tcons, even in the presence of PC61 (Figures 6C,D). Treg expansion reflected the selective pressure of a low-zone IL-2 environment, which increased Treg percentages (Figure 6D) commensurate with increases in FOXP3 expression as assessed by the GFP MFI (Figure 6E). Overall, these data indicate that a defined low concentration range of IL2-CD25 favors outgrowth of FOXP3^+^ Tregs.

**FIGURE 7:**
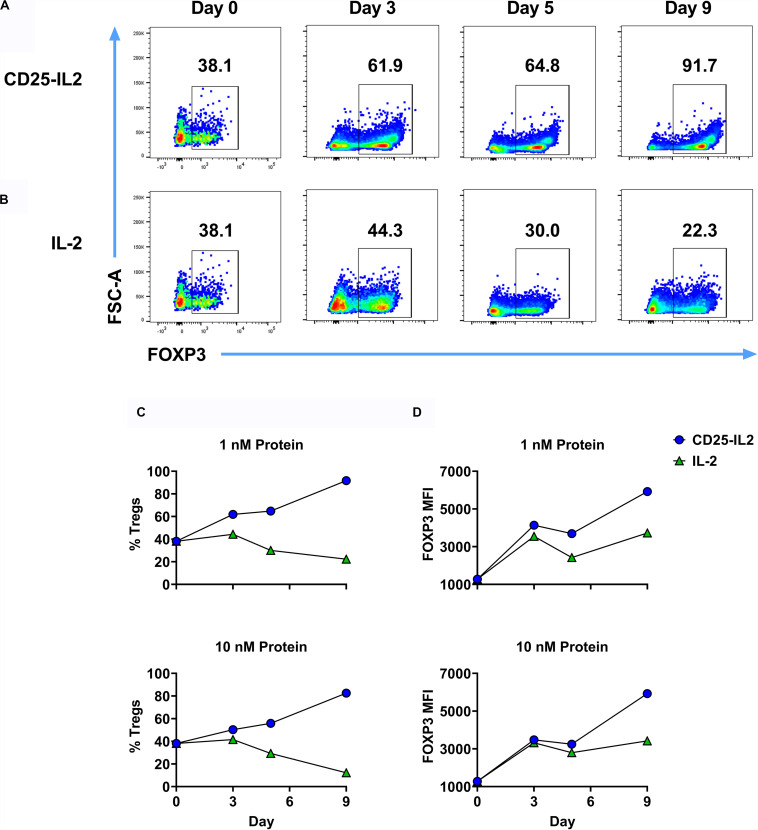
CD25-IL2 enabled dominance of FOXP3^+^ Tregs during continuous culture. 2D2-FIG SPLs were activated at a density of 2 × 10^6^ cells/ml in complete RPMI medium with 1 μM MOG35-55 and 10 nM TGF-β. After 3 days of activation, cells were passaged (designed day 0) at a density of 10^6^ cells/ml with 1 nM CD25-IL2 **(A)** or 1 nM mouse IL-2 **(B)**. Cells were passaged in these conditions every 3–4 days and were analyzed for percentages of CD4^+^ FOXP3^+^ T cells on designated days. Shown are percentages of CD4^+^ FOXP3^+^ Tregs **(C)** and the FOXP3 MFI (CD4^+^ FOXP3^+^ gate) **(D)** when propagated with 1 nM (top) or 10 nM (bottom) of CD25-IL2 or IL-2. These data represent three independent experiments.

Like IL2-CD25, CD25-IL2 also enabled the selection of Treg monocultures (Figure 7). 2D2-FIG SPLs were activated for 3 days with 1 μM MOG35-55 and 10 nM TGF-β to generate Tregs, which were then cultured with 1 nM CD25-IL2 (Figure 7A) or 1 nM IL-2 (Figure 7B) for the designated durations with passages every 3–4 days. Mixed Treg/Tcon lines cultured with CD25-IL2 showed consistent enrichment of FOXP3^+^ Tregs as measured by CD4^+^ FOXP3^+^ Treg percentages (Figure 7C) or FOXP3 MFI (Figure 7D). These data revealed that CD25-IL2 also enabled the dominant outgrowth and stability of FOXP3^+^ Tregs during continuous culture.

### The FPs Maintained Functional and Phenotypic Stability of Suppressive FOXP3^+^ Tregs During Long-Term Culture

**FIGURE 8:**
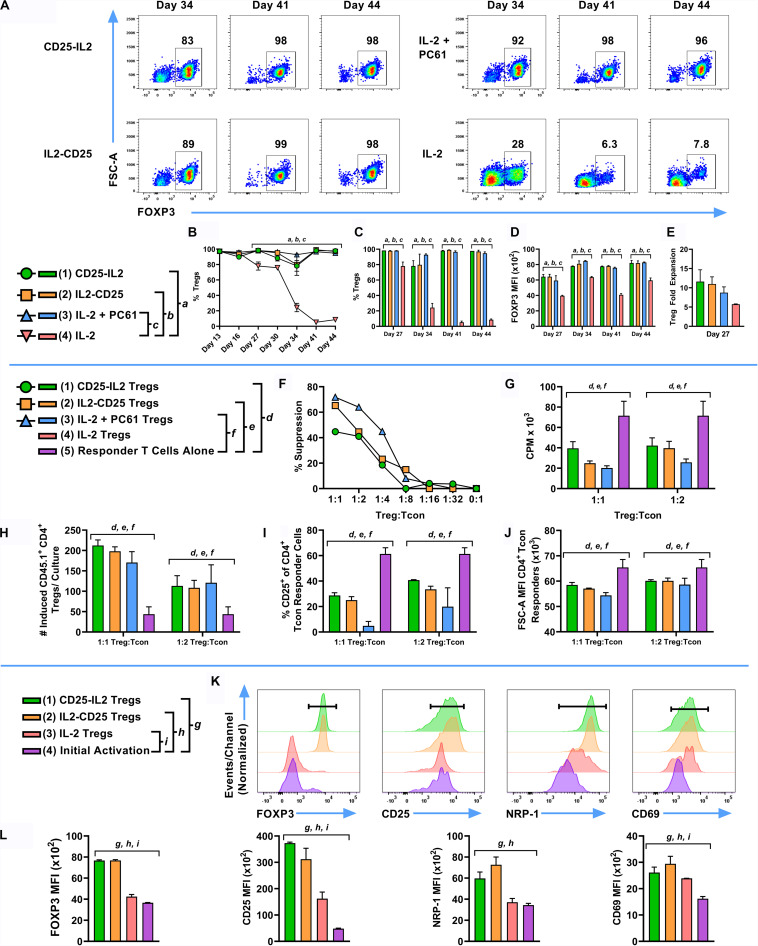
FP-selected Tregs exhibited suppressive activity *in vitro* and displayed stable expression of canonical Treg-associated markers. An essentially pure line of CD45.2 2D2-FIG Tregs was derived by an initial 3-day activation with MOG35-55 and TGF-β followed by continuous propagation in IL2-CD25 for 9 days. These Tregs were then reactivated for 3 days with irradiated DCs and IL-2 in the presence of 1 μM MOG35-55, and 10 nM TGF-β. On day 13 of continuous culture, activated Tregs were passaged (*n* = 3) at a density of 10^6^ cells/ml with 1 nM mouse CD25-IL2, 10 nM IL2-CD25, 1 nM IL-2 and 10 μg/ml PC61 mAb, or 1 nM IL-2. Tregs were passaged in these conditions every 3–4 days and were analyzed for percentages of CD4^+^ FOXP3^+^ T cells on designated days. Shown are the percentages of CD4^+^ FOXP3^+^ Tregs from day 13 through day 44 **(A–C)** with the corresponding the FOXP3 MFI (CD4^+^ FOXP3^+^ gate) **(D)**. On day 23, Tregs were reactivated for 4 days with IL-2, irradiated DCs, MOG35-55, and TGF-β. The average yield from these activation cultures are shown in **(E)**. To test suppressive function, Tregs (day 23) were cultured at various ratios with 25,000 naïve 2D2-FIG CD4^+^ responder T cells and 50,000 irradiated C57BL/6 SPLs for 3 days with 1 μM MOG35-55. Cultures were pulsed with [^3^H]thymidine during the last 24 h of a 3-day culture and were harvested to measure cellular proliferation. Shown are the percent suppression of the CD4^+^ responder T cells **(F)** and the CPM **(G)** at indicated Treg:Tcon ratios. On day 34, CD45.2 2D2-FIG Tregs were co-cultured with 50,000 naïve CD45.1 2D2-FIG CD4^+^ responder T cells and 10,000 irradiated DCs for 3 days with 1 μM MOG35-55. Shown are the number of induced CD45.1^+^ CD4^+^ Tregs among responder T cells (CD3^+^ CD4^+^ CD45.1^+^ gate) **(H)**, percentages of CD25^+^ Tcon responders (CD3^+^ CD4^+^ CD45.1^+^ FOXP3^–^ gate) **(I)**, and the forward scatter (FSC-A/cellular size) MFI of Tcon responders (CD3^+^ CD4^+^ CD45.1^+^ FOXP3^–^ gate) **(J)**. On day 37, Tregs were reactivated with irradiated DCs in the presence of IL-2, 1 μM MOG35-55, and 10 nM TGF-β. As a control, naïve 2D2-FIG SPLs were subjected to an initial activation with 1 μM MOG35-55 and 10 nM TGF-β to generate nascent FOXP3^+^ Tregs. After 4 days of activation (day 41), T cells derived by culture in CD25-IL2, IL2-CD25, or IL-2 were compared to nascent Tregs generated during an initial activation for expression of FOXP3, CD25, NRP-1, and CD69 **(K,L)**. Representative histograms are shown **(K)** for CD4^+^ T cells along with relative MFIs for FOXP3^+^ Tregs **(L)**. **(B–E)** Statistical significance was analyzed by use of a one-way ANOVA with the Holm-Sidak multiple comparisons test comparing each group mean to the group 4 (IL-2) mean. Statistically significant (*p* < 0.05) differences were noted as indicated (*a*, 1 vs 4) (*b*, 2 vs 4) (c, 3 vs 4). **(F–J)** Statistical significance was analyzed by use of a one-way ANOVA with the Holm-Sidak multiple comparisons test comparing each group mean to the group 5 (Responder T Cells Alone) mean. Statistically significant (*p* < 0.05) differences were noted as indicated (*d*, 1 vs 5) (*e*, 2 vs 5) (*f*, 3 vs 5). **(L)** Statistical significance was analyzed by use of a one-way ANOVA with the Holm-Sidak multiple comparisons test comparing each group mean to the group 4 (Initial Activation) mean. Statistically significant (*p* < 0.05) differences were noted as indicated (*g*, 1 vs 4) (*h*, 2 vs 4) (*i*, 3 vs 4). Error bars represent SD. These data represent three independent experiments.

Given that CD25-IL2 and IL2-CD25 FPs positively selected FOXP3^+^ Tregs in culture, an important question was whether these FPs were required to maintain Treg homogeneity during subsequent propagation. To address this question, an established Treg line was split on day 13 into cultures containing 1 nM CD25-IL2, 10 nM IL2-CD25, 1 nM IL-2 and 10 μg/ml PC61 mAb, or 1 nM IL-2 and passaged every 3–4 days in the same conditions with periodic re-activations in the presence of irradiated DCs, MOG35-55, and TGF-β. Cultures maintained in CD25-IL2, IL2-CD25, or IL-2/PC61 sustained essentially homogeneous Treg lines through day 44 whereas Tregs cultured in IL-2 alone exhibited destabilization and were overgrown by Tcons as noted by decreased Treg percentages by day 44 (i.e., ∼8% Tregs) (Figures 8A–C). The FPs and “IL-2 + PC61” not only preserved Treg identity but also promoted high levels of FOXP3 expression as assessed by GFP MFI (Figure 8D). During *in vitro* re-activation (days 23–26), Tregs exhibited an approximate 5–10-fold expansion in numbers (Figure 8E). To test suppressive function, FOXP3^+^ Tregs were mixed with naïve CD4^+^ responder T cells and were cultured with irradiated splenocytes and MOG35-55 for 3 days. These Tregs robustly inhibited MOG-stimulated proliferation of responder CD4^+^ T cell as measured by proliferation (Figures 8F,G). The CD45.2 Tregs also elicited increased numbers of CD45.1 FOXP3^+^ Tregs in the responder T cell population (Figure 8H) while suppressing CD25 expression and cell size enlargement among the responder Tcon population (Figures 8I,J). Previous studies focusing on Tregs cultivated in low IL-2 concentrations and high PC61 concentrations showed a progressively more robust Treg phenotype as a function of time during long-term continuous culture (30). Likewise, activated Tregs that were derived by long-term continuous culture in the presence of CD25-IL2 or IL2-CD25 exhibited higher levels of FOXP3, CD25, NRP-1, and CD69 than activated nascent Tregs isolated from an initial activation with MOG35-55 and TGF-β (Figures 8K,L). These data indicate that continuous culture in the FPs not only enabled stable expansion and outgrowth of FOXP3^+^ Tregs but that these FPs were needed to maintain Treg functional and phenotypic identity during long-term culture.

### CD25-IL2 FP Augmented the Percentages of FOXP3^+^ Tregs *in vivo*

**FIGURE 9:**
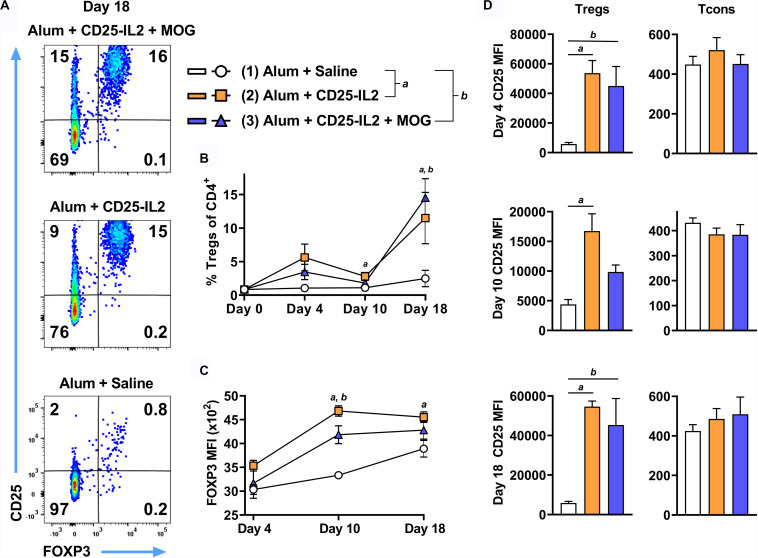
CD25-IL2 FP augmented the percentages of FOXP3^+^ Tregs *in vivo*. On days 0 and 14, 2D2-FIG mice (*n* = 4/group) were vaccinated with saline, CD25-IL2 alone, or the combination of CD25-IL2 and MOG35-55. All injections were administered subcutaneously in Alum. Vaccinations included 3 or 2 nmoles FP (day 0 and 14, respectively) and 4 nmoles MOG35-55. PBMCs were assayed for CD3, CD4, GFP (FOXP3), CD25, and Vβ11 by flow cytometry on days 4, 10, and 18. Shown are **(A)** representative dot plots (day 18 time-point) from each treatment group gated on CD3^+^ CD4^+^ T cells and analyzed for CD25 and FOXP3 expression, **(B)** percentages of FOXP3^+^ Tregs (CD3^+^ CD4^+^ T cell gate) before vaccination (day 0) and for the day 4, 10, and 18 time points, **(C)** the FOXP3 MFIs (CD3^+^ CD4^+^ FOXP3^+^ Treg gate), and **(D)** the CD25 MFIs on Tregs (left, CD3^+^ CD4^+^ FOXP3^+^ gate) and Tcons (right, CD3^+^ CD4^+^ FOXP3^–^ gate). **(D)** Note the use of different *y*-axis scales. Statistical significance was analyzed by a one-way ANOVA with the Holm-Sidak multiple comparisons test. Statistically significant (*p* < 0.05) differences were noted as indicated (*a*, 1 vs 2) (*b*, 1 vs 3). Error bars represent SEM.

These FPs also have potential for increasing Treg activity and numbers *in vivo* and may be useful for boosting tolerogenic vaccination by eliciting the expansion of antigen-specific CD25^high^ Tregs. To assess whether CD25-IL2 acts in synergy with MOG35-55 *in vivo*, 2D2-FIG mice were subcutaneously vaccinated with CD25-IL2 in the Alum adjuvant or were vaccinated with the combination of CD25-IL2 and MOG35-55 in Alum on days 0 and 14 (Figure 9). Mice injected with saline/Alum were used as negative controls. Circulating Tregs were assessed on days 4, 10, and 18. Representative dotplots of CD25 and FOXP3 expression on CD3^+^ CD4^+^ T cells are shown for day 18 (Figure 9A). Vaccination on day 0 with CD25-IL2 caused an increase in Treg percentages and CD25 MFI on Tregs by day 4, but these values waned by day 10, whereas the boost on day 14 caused a robust rebound of CD25^high^ FOXP3^+^ Tregs by day 18 (Figures 9B,D) (note different *y*-axis scales in Figure 9D). The initial vaccination and the subsequent boost elicited elevated CD25 expression on Tregs but not on Tcons (Figure 9D). The initial vaccination also elicited increases in FOXP3 expression on a per cell basis, which provided evidence of activated stable Tregs (Figure 9C). The mechanism was driven by low-zone IL-2 activity rather than antigen because Treg responses were only modestly affected by the presence or absence of MOG35-55. These data indicated that CD25-IL2 had robust Treg expansive activities *in vivo*, in that two injections of CD25-IL2/Alum resulted in greater than an approximate 15-fold increase in percentages of circulating, stable CD25^high^ FOXP3^+^ Tregs without affecting CD25 expression by Tcons.

**FIGURE 10:**
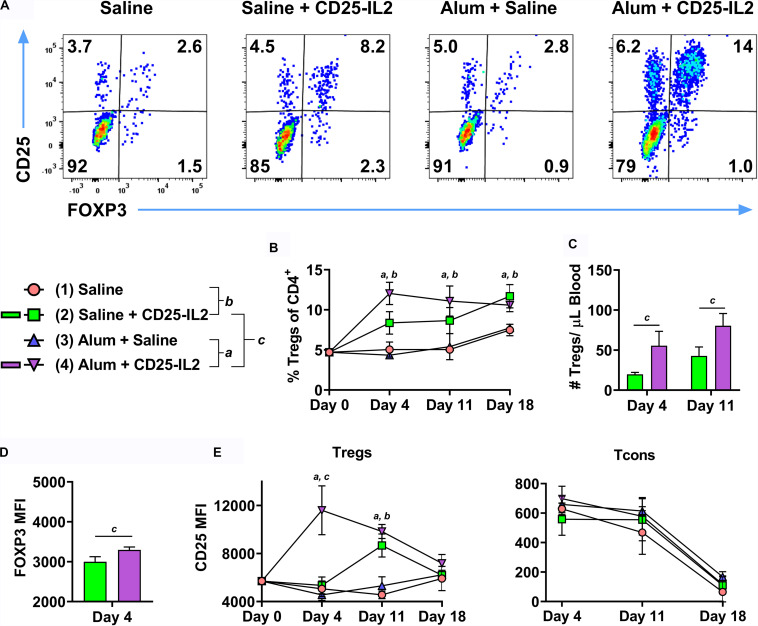
The Alum adjuvant augmented Treg responses to CD25-IL2 *in vivo*. On day 0, FIG mice (*n* = 4/group) were vaccinated with (1) saline, (2) CD25-IL2 in saline, (3) saline in Alum, or (4) CD25-IL2 in Alum. All injections were subcutaneous at a dose of 3 nmoles. Groups were boosted with the same vaccine on days 7 and 14. PBMCs were assayed for CD3, CD4, GFP (FOXP3), and CD25 by flow cytometry on days 4, 11, and 18. Shown are **(A)** representative dot plots (day 4 time-point) from each treatment group gated on CD3^+^ CD4^+^ T cells and analyzed for CD25 and FOXP3 expression and **(B)** percentages of FOXP3^+^ Tregs (CD3^+^ CD4^+^ T cell gate) before vaccination (day 0) and for the day 4, 11, and 18 time-points. Shown **(C)** are absolute numbers of FOXP3^+^ Tregs (CD3^+^ CD4^+^ T cell gate) for the day 4 and 11 time-points and **(D)** the FOXP3 MFIs (CD3^+^ CD4^+^ FOXP3^+^ gate) for the day 4 time-point. Shown **(E)** are CD25 MFIs of the Treg subset (left, CD3^+^ CD4^+^ FOXP3^+^ gate) and Tcon subset (right, CD3^+^ CD4^+^ FOXP3^–^ gate). Note use of different *y*-axis scales. Statistical significance (*p* < 0.05) was analyzed by use of a *t*-test as indicated (*a*, 3 vs 4) (*b*, 1 vs 2) (c, 2 vs 4). Error bars represent SEM.

Because the Alum adjuvant was used in all groups in Figure 9, an important issue was whether Alum contributed to an IL-2 dependent, antigen-independent Treg response, particularly in the framework of a wild type TCR repertoire. To assess this issue, FIG mice were vaccinated subcutaneously with CD25-IL2 in Alum or saline on day 0 and were boosted on days 7 and 14 (Figure 10). Two additional control groups included “saline in Alum” and “saline alone.” Representative dot plots of CD3^+^ CD4^+^ T cells are shown for the day 4 time-point (Figure 10A). CD25-IL2 in saline or Alum elicited higher Treg percentages on days 4, 11, and 18 compared to respective groups without the FP (Figure 10B). The Alum adjuvant potentiated CD25-IL2 activity and Treg percentages on day 4 but not subsequently on days 11 or 18. The Alum adjuvant also augmented CD25-IL2 activity as measured by circulating numbers of Tregs on day 4 and 11 (Figure 10C), increased FOXP3 MFI on day 4 (Figure 10D), and elevated CD25 expression on Tregs on day 4 but not thereafter (Figure 10E, left). The Alum adjuvant had no independent activity on Tregs in the absence of CD25-IL2. The actions of CD25-IL2 in Alum were restricted to Tregs and were without effect on Tcons (Figure 10E, right). Overall, these data reveal that Alum acutely augments the Treg-selective activity of CD25-IL2, particularly by driving synergistic increases in FOXP3 and CD25 expression on circulating Tregs.

### Tolerogenic Vaccination and CD25-IL2 Acted Synergistically to Induce and Maintain Tregs *in vivo*

**FIGURE 11:**
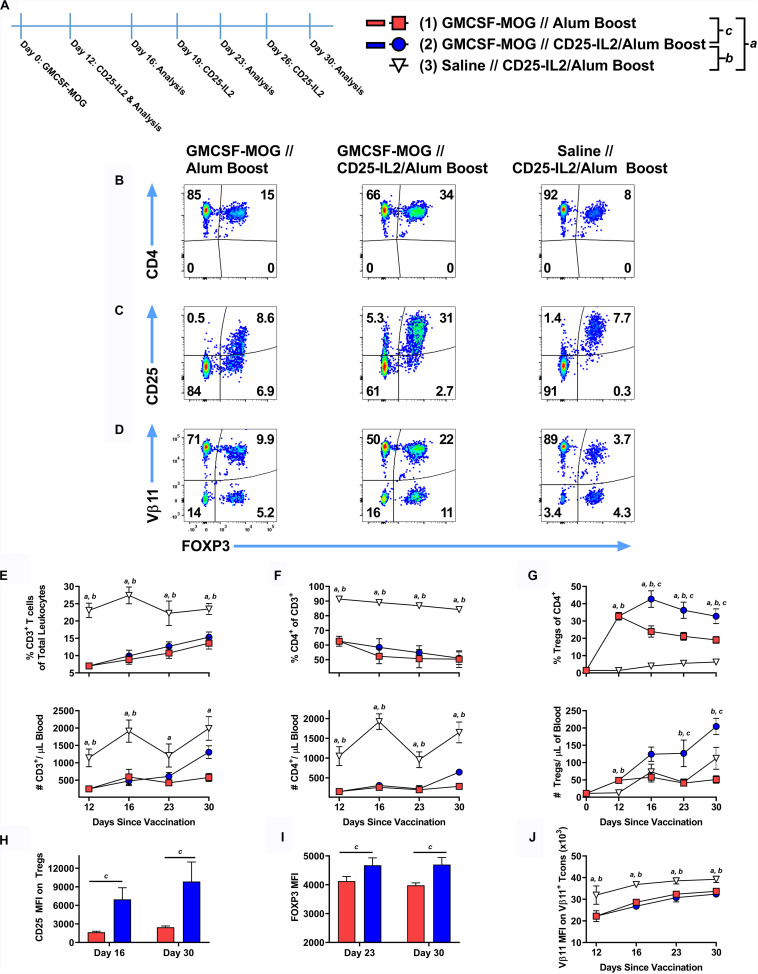
After tolerogenic vaccination, boosting with CD25-IL2/Alum maintained Treg responses *in vivo*. On day 0, 2D2-FIG mice were subcutaneously vaccinated with 4 nmoles of the tolerogenic vaccine GMCSF-MOG (in saline) or saline alone. On days 12, 19, and 26, mice were boosted with either saline in Alum or 3 nmoles CD25-IL2 in Alum (*n* = 6–8/group). PBMCs were assayed for CD3, CD4, GFP (FOXP3), CD25, and Vβ11 by flow cytometry on days 12, 16, 23, and 30. Shown **(A)** is a timeline of the boosting schedule and the PBMC analysis. Shown are representative dot plots (day 30 time-point) from each treatment group gated on CD3^+^ CD4^+^ T cells and analyzed for **(B)** CD4, **(C)** CD25, and **(D)** Vβ11 and FOXP3 expression. Shown are the percentages (top row) and total numbers (bottom row) of **(E)** CD3^+^ T cells, **(F)** CD4^+^ T cells (CD3^+^ gate), and **(G)** FOXP3^+^ Tregs (CD3^+^ CD4^+^ gate) on days 12, 16, 23, and 30 time-points. **(H)** The CD25 MFIs on the CD3^+^ CD4^+^ FOXP3^+^ Tregs are shown for the day 16 and 30 time-points. **(I)** The FOXP3 MFIs on the CD3^+^ CD4^+^ FOXP3^+^ Tregs are shown for the day 23 and 30 time-points. **(J)** The TCR-Vβ11 MFIs are shown for CD3^+^ CD4^+^ Vβ11^+^ FOXP3^–^ Tcon cells. Statistical significance was analyzed by use of a one-way ANOVA with the Holm-Sidak multiple comparisons test. Statistically significant (*p* < 0.05) differences were noted as indicated (*a*, 1 vs 3), (*b*, 2 vs 3), (c, 1 vs 2). Error bars represent SEM.

These findings raised the prospect that CD25-IL2 may have synergistic utility with tolerogenic vaccine regimens. To test this possibility, 2D2-FIG mice were subcutaneously vaccinated with the tolerogenic vaccine GMCSF-MOG (in saline) or with saline alone on day 0 and were boosted on days 12, 19, and 26 with “CD25-IL2 in Alum” or “saline in Alum” (Figure 11A). Representative dotplots of CD3^+^ CD4^+^ T cells are shown for the day 30 analysis (Figures 11B–D). As previously shown (20), GMCSF-MOG caused a sustained loss of CD3^+^ CD4^+^ T cells (Figures 11E,F) coupled with a robust expansion of MOG-specific Tregs that peaked by day 12 (Figure 11G). From the peak at day 12, GMCSF-MOG-induced Tregs waned through day 30 whereas boosting with CD25-IL2 in Alum prevented attenuation of Treg levels and sustained high levels of circulating Tregs throughout the time course (Figure 11G). The combination of tolerogenic vaccination and boosting with CD25-IL2 was associated with high levels of CD25 and FOXP3 on Tregs through day 30 (Figures 11H,I). GMCSF-MOG also caused downregulation of TCR as shown by decrements in Vβ11 expression on Vb11^+^ Tcons (Figure 11J) by a mechanism that was unaffected by boosting with CD25-IL2 in Alum. These findings indicate that tolerogenic vaccine responses were sustained by “CD25-IL2 in Alum” boosters, which maintained high circulating levels of vaccine-induced CD25^high^ FOXP3^+^ Tregs.

### CD25-IL2 and IL2-CD25 FPs Lacked Treg-Selective Anti-inflammatory Activity in IL-2 Replete Environments

**FIGURE 12:**
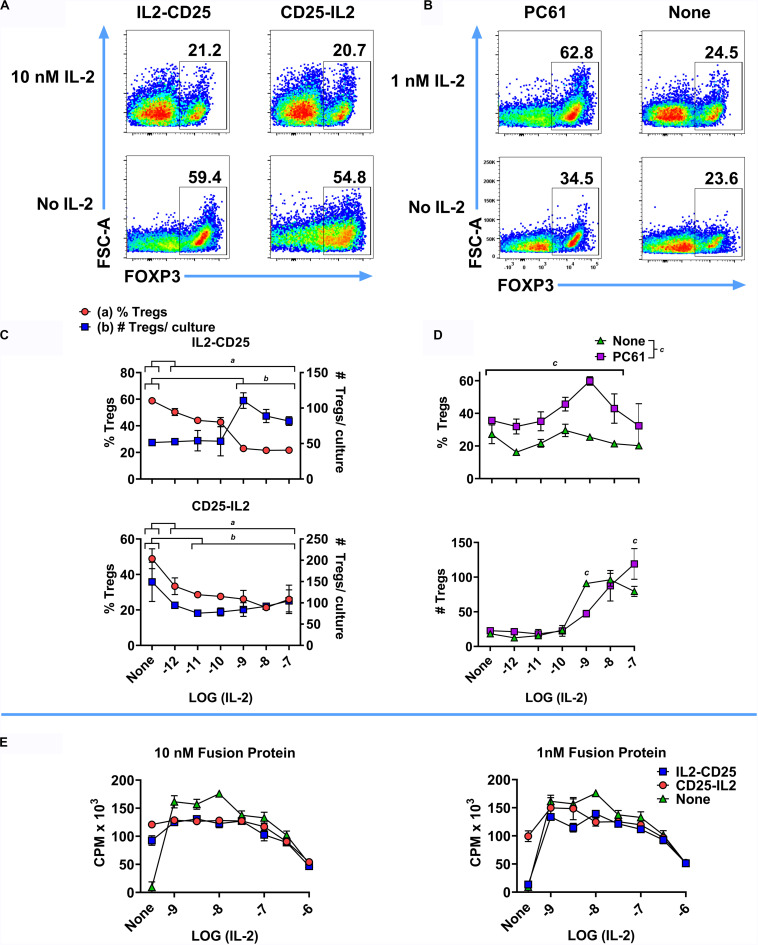
CD25-IL2 and IL2-CD25 FPs lacked Treg-selective activity in IL-2 replete environments. 2D2-FIG SPLs were activated for 3 days at a density of 2 × 10^6^ cells/ml in complete RPMI medium with 1 μM MOG35-55 and 10 nM TGF-β to generate a mixed Treg/Tcon line (approximately 50%/50%, respectively). **(A–D)** These T cells were then passaged (designated day 0) at a density of 1 × 10^6^ cells/ml with or without 10 nM IL2-CD25, 10 nM CD25-IL2, or 10 μg/ml PC61 in the presence or absence of designated concentrations of mouse IL-2 for 6 days and were analyzed for FOXP3 expression. Shown are representative dot plots analyzed for FOXP3 expression **(A,B)** together with Treg percentages and numbers **(C,D)** at designated IL-2 concentrations (*x*-axis). **(C)** The group mean for each IL-2 concentration was compared to the mean of control samples lacking IL-2 for each FP. Statistically significant differences (*p* < 0.05) were noted as indicated (*a*, % Tregs and *b*, # Tregs/culture). **(D)** The effect of PC61 was analyzed by comparing the group mean for each *x*-axis value in the presence or absence of PC61. **(E)** A transformed, IL-2 dependent mouse T cell line (SJL-PLP.1) was cultured at 5,000 cells/well with designated concentrations of purified mouse IL-2 (*x*-axis) with or without 10 nM or 1 nM CD25-IL2 or IL2-CD25. Cultures were pulsed with [^3^H]thymidine during the last 24 h of a 3-day culture and were harvested to measure cellular proliferation. Error bars represent the SD.

The CD25-IL2 and IL2-CD25 FPs had intrinsic IL-2 activity but were not predicted to antagonize exogenous sources of free IL-2 and were not predicted to impose Treg-conducive low-zone IL-2 signaling in environments bearing high concentrations of free IL-2. Thus, these FPs have Treg-inductive activity in the absence of free IL-2 and Treg-conducive activity in quiescent *in vivo* environments in the absence of exogenous IL-2 (Figures 5–11), but these FPs are predicted to lack Treg-biasing activities in inflamed tissues or in environments replete in extrinsic IL-2. To assess the effectiveness of these FPs in the presence of IL-2 *in vitro*, a mixed Treg/Tcon line was cultured for 6 days with or without 10 nM IL2-CD25, 10 nM CD25-IL2, or 10 μg/ml PC61 in the presence or absence of designated concentrations of mouse IL-2 (Figures 12A–D). Representative dotplots of FOXP3 expression in the CD4^+^ T cell population are shown in Figures 12A,B. After 6 days of culture, the Treg-selective activities of both FPs were blocked by progressively higher concentrations of IL-2 (Figure 12C). At 10 nM IL2-CD25 or CD25-IL2, progressive increases in IL-2 concentrations (*x*-axis) caused a diminution of Treg percentages via IL-2 dependent outgrowth of Tcons relative to modest changes in the absolute numbers of Tregs (Figure 12C). At 10 μg/ml PC61, 1 nM IL-2 caused a peak in Treg percentages, but higher IL-2 concentrations (10–100 nM) overshadowed increased Treg proliferation with decreased Treg percentages due to more rapid growth of Tcons (Figure 12D). In [^3^H]thymidine incorporation experiments, the FPs had modest inhibitory activity at low IL-2 concentrations (1–10 nM) but had no effect at higher IL-2 concentrations (100 nM or 1 μM) (Figure 12E). The inhibitory activity of FPs at low IL-2 concentrations may be due to the additive action of IL-2, in that the IL-2 activity curve was bell-shaped and additional IL-2 activity above the peak concentration resulted in less T cell growth. Collectively, these data provide evidence that, as expected, these FPs lacked meaningful Treg-conducive activities in environments bearing high concentrations of IL-2.

**FIGURE 13:**
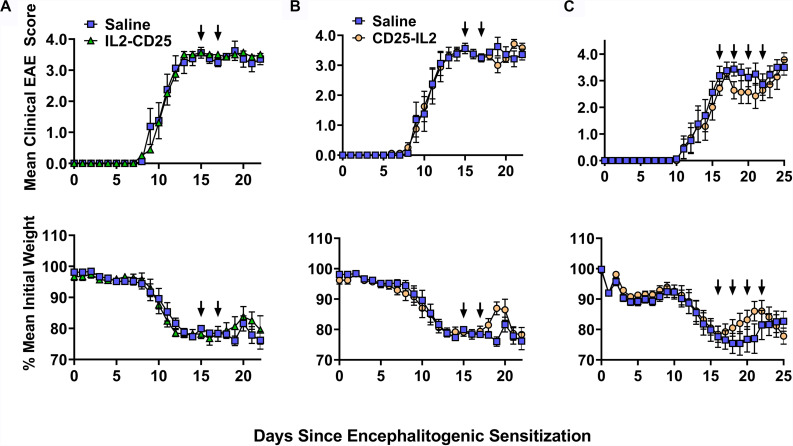
CD25-IL2 did not effectively control EAE in an IL-2 replete environment. On day 0, C57BL/6 mice were immunized with 200 μg MOG35-55 emulsified in CFA. Mice received 400 ng of Pertussis toxin intraperitoneal on days 0 and 2. At peak disease (day 15) mice were immunized with saline (control group) or with 2 nmoles **(A)** IL2-CD25 in saline or **(B)** 2 nmoles CD25-IL2 in saline. Mice were boosted on day 17 with the same formulation. **(C)** At peak disease (day 16) mice were immunized with 2 nmoles CD25-IL2 in saline or with saline alone. Mice were then boosted on days 18, 20, 22 with the same formulation. Shown are the daily mean clinical EAE scores and daily mean initial weight through the end of the experiment. Error bars represent SEM. Arrows indicate the schedule of FP administration.

These FPs also had limited activity as a therapy for EAE. In C57BL/6 mice that were exhibiting severe paralytic signs of EAE, administration of IL2-CD25 lacked therapeutic activity when given during severe paralytic EAE (Figure 13A). CD25-IL2 in saline appeared to have temporary therapeutic activity measured by alleviation of clinical signs and reversal of EAE-associated weight loss (Figures 13B,C). However, these beneficial activities were modest and were lacking in statistical and biological significance. Cessation of FP treatment resulted in disease relapse marked by a return to a severe course of chronic EAE and severe cachexic weight loss. Similar therapeutic regimens were used previously to show that tolerogenic vaccines effectively reversed the chronic phase of EAE and elicited remission (16, 19, 37). Overall, these data indicate that these FPs have limited utility when administered in IL-2 replete inflammatory environments, which may include environments marked by chronic inflammation and autoimmunity.

## Discussion

### CD25-IL2 and IL2-CD25 FPs Enable the Preferential Expansion of CD25^high^ FOXP3^+^ Tregs

These FPs were designed to establish a constrained low-zone IL-2 signaling window that would preferentially expand Tregs *in vitro* and enhance Treg responses *in vivo*. This study provided several novel lines of evidence that CD25-IL2 and IL2-CD25 FPs exhibited low-zone IL-2 signaling intensities conducive for selective Treg responses. First, this study revealed that these FPs exhibited IL-2 activity with a potency substantially reduced compared to IL-2 (Figure 1). These FPs assembled into higher MW multimers with occluded IL-2 binding sites that were in equilibrium with conformers having exposed IL-2 domains, the latter of which signaled upon binding transmembrane CD25 on T cells via an intact IL2Rαβγ (Figures 2–4). Second, this study uniquely revealed that these FPs can be used to derive essentially pure lines of FOXP3^+^ Tregs *in vitro* (Figures 5–8). Bell-shaped concentration response curves were revealed in which a window of FP concentrations enabled stable dominant Treg overgrowth of Tcons to yield continuous primary lines comprised of >95% Tregs that expressed suppressive function and stable canonical Treg-associated markers (Figure 8). These FPs therefore may be useful for the *in vitro* expansion of pure primary Treg populations advantageous for staging autologous Treg-based adoptive immunotherapy protocols. Third, this study revealed that these FPs can be used *in vivo* to increase the presence of circulating CD25^high^ Tregs (Figures 9, 10). This study also uniquely revealed that these FPs can be used in concert with myelin-specific tolerogenic vaccination in which a single subcutaneous injection of the vaccine elicits ∼30% Tregs in a TCR-transgenic model (Figure 11). In concert with this tolerogenic vaccine, these FPs prolonged circulating levels of MOG-specific Tregs over a 30-day observation window (Figure 11). This new insight thereby addresses a major problem in that Treg interventional therapies elicit Treg responses that wane over time, but these FPs can be used to prolong the longevity of the Treg response at optimal levels. These FPs, however, did not affect numbers or phenotypes of Tcon subsets. Fourth, this study uniquely showed that inclusion of the FPs in the Alum adjuvant augmented the induction and maintenance of Tregs *in vivo* (Figure 10). This study is thus the first to show the utility of providing these FPs in the Alum adjuvant, which most likely improves the *in vivo* half-life and tolerogenic efficacy (Figure 10). The primary limitation is that these FPs lacked Treg inductive activity in the presence of high concentrations of free monomeric IL-2 or when administered during an ongoing autoimmune inflammatory attack (Figures 12, 13). This study thereby uniquely advances the field by showing the potential applicability of IL2-CD25 FPs in that this tolerogenic platform can be used to derive CD25^high^ FOXP3^+^ Treg monocultures, facilitate tolerogenic vaccination, and synergistically interact with the Alum adjuvant to elicit heightened Treg responses. Overall, these data show that these FPs enable selective Treg dominance *in vitro* and *in vivo*. Additional research is needed to explore this novel technology in models of autoimmune pathogenesis and human Treg biology.

During the course of this study, another group published findings on an independently derived set of IL2-CD25 FPs as biologics in the context low-dose IL-2 therapy (36). These FPs also had weak IL-2 activity coupled with a more durable *in vivo* half-life compared to IL-2. The primary focus was on a murine IL2-(G3S)_3_-CD25 FP, in which the 12-aa linker favored formation of IL2-CD25 anti-parallel homodimers in dynamic equilibrium with a minor population of IL2-CD25 monomers. The IL2-CD25 homodimers were considered biologically inert whereas the IL2-CD25 monomers were considered biologically active due to a freely accessible IL-2 domain. These FPs compared favorably with IL-2/anti-IL-2 antibody complexes *in vivo* for the temporary expansion of FOXP3^+^ CD25^high^ Tregs. Thus, two independent studies [(36) and Figures 9, 10] have shown the utility of IL2-CD25 FPs for use in selectively expanding Tregs *in vivo* with no commensurate action on naïve or effector/memory Tcon subsets. The 23-aa linker used in our study was substantially longer than the 12-aa linker used in (36). A longer 23-aa linker may provide greater degrees of freedom conducive for assembly of higher-order multimeric protein structures. Indeed, our protein analyses suggested that CD25-IL2 and IL2-CD25 were primarily multimeric rather than monomeric or dimeric in size (Figure 4). Thus, the length of the CD25-IL2 linker may determine the complexities of intermolecular interactions between the two domains and the ultimate multimer size.

### The CD25-IL2/IL2-CD25 FPs Provide an Enduring Steady-State Supply of Low-Zone IL-2

The concept that an IL-2-accessible conformer represented the active form was consistent with three lines of evidence. First, the FPs lacked IL-2 stimulatory activity in assays of CD25^–/–^ T cells (Figure 1). These data indicated that FPs required transmembrane CD25 for productive signaling. If the FPs interacted directly with IL2Rβγ, then the FP would predictably have greater potency compared to IL-2 alone, because the CD25 FP domain should contribute affinity to a FP:IL2Rβγ interaction. However, the data discounted this possibility. Second, the FPs bound to HEK cells that expressed full length murine CD25 but lacked IL2Rβγ (Figure 2). These data indicated that transmembrane CD25 was sufficient for FP binding. Third, the FPs catalyzed the dominant outgrowth of Tregs from mixed Treg/Tcon lines (Figures 5–8). These data also indicate that surface CD25 was instrumental for FP action, because the canonical attribute of Tregs is higher levels of CD25 expression compared to Tcons rather than differential IL2Rβγ expression compared to Tcons.

The selectivity of these FPs for Tregs may suggest more complicated signaling models. For example, the unencumbered FP-IL2 domain may interact with transmembrane CD25 to initiate signaling on T cells via the IL2Rαβγ complex whereas the tethered FP-CD25 domain may rapidly recapture the FP-IL2 domain from the transmembrane FP-IL2/IL2Rαβγ complex to terminate signaling. Thus, the FP-CD25 domain may not only quantitatively decrease the availability of FP-IL2 domain via sequestration, the FP-CD25 domain may qualitatively alter the kinetic lifetime of a productive FP-IL2: transmembrane IL-2Rαβγ complex. That is, free IL-2 may elicit sustained signaling through the IL-2Rαβγ receptor whereas CD25-IL2 may result in rapid on-off kinetics due to competition between the tethered FP-CD25 domain and transmembrane CD25 for the FP-IL2 domain. According to this scenario, CD25-IL2 would attain productive signaling thresholds only when high surface concentrations of transmembrane CD25 drove the equilibrium away from the occluded FP conformer and toward the FP-IL2: IL-2Rαβγ complex, which would only occur on the surface of FOXP3^+^ CD25^high^ Tregs. In addition, if FPs promote rapid on-off kinetics of the FP-IL2 domain with the IL2Rαβγ complex, FPs may escape internalization and degradation and thereby may have a longer half-life to mediate chronic low-zone IL-2 signaling.

Regarding Treg-selective activities *in vitro*, the CD25-IL2 and IL2-CD25 FPs facilitated Treg competitive dominance only within the confines of a defined concentration range. FP concentrations below this range were insufficient to sustain T cell growth and survival. FP concentrations that exceeded this range promoted excessive IL-2 signaling and consequently Tcon dominance rather than Treg dominance (Figure 5). Aside from these quantitative considerations, the FPs may also have qualitative activities distinct from those of free IL-2. The effective window of FP concentrations that provided robust selection of Tregs was broad and reliable. In contrast, IL-2 did not have a feasible window for selection and expansion of Tregs because the extremely low concentrations of IL-2 that were adequate for selecting Tregs were also insufficient for growth and expansion of T cells *in vitro* (Figure 5). Therefore, imposing a low-zone IL-2 window with IL-2 alone was not feasible in our culture systems. An important consideration is that the FPs may exist as an inert reservoir to provide a buffered equilibrium that maintains low-zone IL-2 across time, thereby maintaining steady-state supplies of low-zone IL-2 despite constant consumption of free IL-2 by T cells.

### CD25-IL2/IL2-CD25 FPs and Alum Are Synergistic Tolerogenic Adjuvants

Fusion proteins included in the Alum adjuvant had higher Treg selective activities *in vivo* (Figure 10) even though the Alum adjuvant facilitates a concentrated repository effect at the inoculation site and in the lymphatic drainage. Possibly, binding of the FP to Alum may restrain the acute availability of the FP-IL2 while simultaneously prolonging availability over time. This finding (Figure 10) reinforced the concept that the Alum adjuvant was fully compatible with tolerogenic vaccination and Treg responses. For example, Alum was required in tolerogenic vaccines comprised of IFN-β and neuroantigen for Treg inductive responses and tolerance induction (37). Both saline and Alum were permissive vehicles for GMCSF-neuroantigen tolerogenic vaccines, in that subcutaneous administration of vaccines formulated in either saline or Alum elicited high levels of circulating FOXP3^+^ Tregs (20).

Because naked protein/peptide antigens are inefficient tolerogens, the field needs to identify adjuvants that amplify or at least permit tolerogenic vaccination. This study adds to the accumulating evidence that both CD25-IL2/IL2-CD25 FPs and Alum are efficacious adjuvants that can be used to optimize tolerogenic vaccination. Whereas other parameters such as inefficient self-antigens or immunoregulatory cytokines direct a vaccine toward tolerogenic outcomes, an adjuvant is the multiplier that amplifies that outcome. Thus, Alum is permissive for either immunogenic or tolerogenic outcomes depending on other components in the vaccine. Alum promotes immunogenic responses to foreign antigens but promotes tolerogenic outcomes when coupled with inefficient self-antigens or regulatory cytokines such of IFN-β (20, 37). Unlike Alum, which may be considered a permissive multiplier of either immunogenic or tolerogenic outcomes, the CD25-IL2/IL2-CD25 FPs have a pronounced bias toward Treg tolerogenic responses due to low-zone IL-2 signaling. Hence, these FPs have promise as direct Treg-polarizing adjuvants in the future development of tolerogenic vaccines.

### The CD25-IL2/IL2-CD25 FPs Have Limited Tolerogenic Efficacy in IL-2 Replete Environments

A primary limitation is that CD25-IL2 and IL2-CD25 FPs were not intended to compete with free IL-2 and were shown to lack significant antagonistic activity when mixed with free IL-2 in proliferative bioassays (Figure 12E). These FPs mediated Treg-selective activity only in the absence of exogenous sources of IL-2 and lacked Treg-selective activity when mixed with high concentrations of IL-2 *in vitro* (Figures 12A–D). Administration of these FPs during the paralytic phase of EAE did not result in a statistically significant benefit (Figure 13). Rather, the IL2-CD25 FP lack any detectable activity and the CD25-IL2 FP appeared to provide only temporary relief when administered into mice exhibiting severe paralytic signs of EAE, which likely represents an IL-2 replete environment (43, 44). This limitation may restrict the efficacy of FPs for direct clinical application as a low-zone IL-2 therapy in autoimmune disease, because many autoimmune diseases are marked by relatively high IL-2 concentrations in target tissues (44–48). Rather, this study indicates that these FPs are optimal for *in vitro* selection of continuous Treg lines and for expansion of Tregs in quiescent environments *in vivo* because both environments lack exogenous sources of IL-2.

Although these FPs appeared to have marginal inhibitory activity when delivered at the height of a pathogenic autoimmune response in a model of EAE (Figure 13), a similar IL2-CD25 FP prevented autoimmune diabetes when FP administration was initiated before disease onset (36). That is, IL2-CD25 inhibited disease when administered during weeks 6–11 or weeks 12–17 with disease onset at week 14. Thus, these FPs may inhibit antecedent inflammatory responses that subsequently culminate in overt autoimmune disease. Likewise, a previous study focusing on EAE showed that low-dose administration of IL-2 prior to the sensitization phase ameliorated the subsequent expression of disease whereas administration of IL-2 immediately preceding or during disease onset lacked therapeutic activity (49). Thus, it would be of theoretical interest to assess CD25-IL2 and IL2-CD25 FPs earlier in the disease course or as prophylactics before encephalitogenic sensitization in the presence or absence of Alum to assess whether these modalities can control disease induction in IL-2 limited environments before establishment of maximal inflammation.

## Conclusion

CD25-IL2 and IL2-CD25 FPs represent a new class of biologics designed to exploit the low-zone IL-2 signaling niche of FOXP3^+^ CD25^high^ Tregs. This study provides evidence that these FPs can be used to expand essentially pure cultures of stable FOXP3^+^ CD25^high^ Tregs without overgrowth by effector/memory Tcons. These FPs can be used *in vivo*, particularly in combination with the Alum adjuvant, to augment the CD25^high^ Treg-inductive activity of myelin-specific tolerogenic vaccines.

## Data Availability Statement

All datasets generated for this study are included in the article/supplementary material.

## Ethics Statement

The animal study was reviewed and approved by the Institutional Animal Care and Use Committee, East Carolina University.

## Author Contributions

KD and MM designed the project and wrote the manuscript. KD performed most experiments. CM contributed to the experimentation. BG was instrumental in performing and interpreting the protein analysis. All authors analyzed the data, provided intellectual input, and edited the manuscript.

## Conflict of Interest

The authors declare that the research was conducted in the absence of any commercial or financial relationships that could be construed as a potential conflict of interest.

## Abbreviations

aa: amino acid
CD122: IL2R β
CD132: IL2R γ
CD25: IL2R α
CFA: complete Freund’s adjuvant
DC: dendritic cell
EAE: experimental autoimmune encephalomyelitis
FIG: Foxp3-IRES-GFP knock-in allele
FP: fusion protein
GFP: green fluorescent protein
GMCSF-MOG: granulocyte-macrophage colony stimulating factor fused to myelin oligodendrocyte glycoprotein peptide 35-55
HEK: human embryonic kidney cells
mAb: monoclonal antibody
MFI: mean fluorescence intensity
MHC: major histocompatibility complex
MOG: myelin oligodendrocyte glycoprotein
OVA: ovalbumin
PBMC: peripheral blood mononuclear cells
sCD25: soluble CD25
SD: standard deviation
SEM: standard error of the mean
SPL: splenocyte
Tcon: T conventional cell
TCR: T cell receptor
TGF- β: transforming growth factor beta
Treg: FOXP3^+^ regulatory T cell.
